# Capture and Detection of Circulating Glioma Cells Using the Recombinant VAR2CSA Malaria Protein

**DOI:** 10.3390/cells8090998

**Published:** 2019-08-28

**Authors:** Sara R. Bang-Christensen, Rasmus S. Pedersen, Marina A. Pereira, Thomas M. Clausen, Caroline Løppke, Nicolai T. Sand, Theresa D. Ahrens, Amalie M. Jørgensen, Yi Chieh Lim, Louise Goksøyr, Swati Choudhary, Tobias Gustavsson, Robert Dagil, Mads Daugaard, Adam F. Sander, Mathias H. Torp, Max Søgaard, Thor G. Theander, Olga Østrup, Ulrik Lassen, Petra Hamerlik, Ali Salanti, Mette Ø. Agerbæk

**Affiliations:** 1 Centre for Medical Parasitology at Department for Immunology and Microbiology, Faculty of Health and Medical Sciences, University of Copenhagen and Department of Infectious Disease, Copenhagen University Hospital, 2200 Copenhagen, Denmark; 2 VarCT Diagnostics, 2200 Copenhagen, Denmark; 3 Danish Cancer Society Research Center, 2100 Copenhagen, Denmark; 4 Department of Urologic Sciences, University of British Columbia, and Vancouver Prostate Centre, Vancouver, BC V6H 3Z6, Canada; 5 Centre for Genomic Medicine, Copenhagen University Hospital, 2100 Copenhagen, Denmark; 6 ExpreS^2^ion Biotechnologies, SCION-DTU Science Park, 2970 Hørsholm, Denmark; 7 Department of Oncology, Copenhagen University Hospital, 2100 Copenhagen, Denmark

**Keywords:** circulating tumor cells (CTCs), glioma, biomarker, rVAR2, malaria, enrichment and detection technologies

## Abstract

Diffuse gliomas are the most common primary malignant brain tumor. Although extracranial metastases are rarely observed, recent studies have shown the presence of circulating tumor cells (CTCs) in the blood of glioma patients, confirming that a subset of tumor cells are capable of entering the circulation. The isolation and characterization of CTCs could provide a non-invasive method for repeated analysis of the mutational and phenotypic state of the tumor during the course of disease. However, the efficient detection of glioma CTCs has proven to be challenging due to the lack of consistently expressed tumor markers and high inter- and intra-tumor heterogeneity. Thus, for this field to progress, an omnipresent but specific marker of glioma CTCs is required. In this article, we demonstrate how the recombinant malaria VAR2CSA protein (rVAR2) can be used for the capture and detection of glioma cell lines that are spiked into blood through binding to a cancer-specific oncofetal chondroitin sulfate (ofCS). When using rVAR2 pull-down from glioma cells, we identified a panel of proteoglycans, known to be essential for glioma progression. Finally, the clinical feasibility of this work is supported by the rVAR2-based isolation and detection of CTCs from glioma patient blood samples, which highlights ofCS as a potential clinical target for CTC isolation.

## 1. Introduction

Diffuse gliomas are the most common primary malignant brain tumors [1]. As the name implies, a general trait of these tumors is their diffuse invasion into the brain parenchyma, which impedes complete surgical resection and most likely explains the poor prognosis and frequent local recurrence [2]. A precise classification of diffuse gliomas is needed for the optimal diagnosis, stratification, and treatment of patients [3,4]. During the past decades, technologies for biopsy-based classification of diffuse gliomas have increased in their complexity [5,6,7]. However, repeated access to information regarding tumor progression remains challenging, due to the risk and inconvenience that are associated with performing patient brain biopsies. Several studies indicate that the cells constituting the infiltrative and invasive front of gliomas harbor tumor-initiating capacity and may be responsible for drug resistance and tumor recurrence [8,9,10]. Most likely, these migrating cells would also be the ones accessing the blood stream. Therefore, the isolation of circulating tumor cells (CTCs) from a liquid biopsy, such as blood or cerebrospinal fluid, could provide non-invasive, repeatable access to primary glioma cells for molecular analysis. 

Tumors of the central nervous system were until recently not considered to be metastatic. However, organ recipients receiving organs from patients who succumbed to glioblastoma multiforme (GBM) have developed extracranial metastases, which strongly suggests that these organs harbored disseminated GBM cells [11]. In line with this, a few studies using different isolation and detection methods have detected CTCs in blood from glioma patients [12,13,14,15]. Taken together, these studies provide evidence that invasive glioma cells successfully intravasate to the blood circulation and may therefore potentially become an important and easily available source of information on the mutational and phenotypic state of the primary tumor. Molecular analysis of the circulating glioma cells could provide basis for the design and monitoring of personalized treatment strategies, as it has been the case with breast, prostate, and lung cancer [16,17,18]. However, the high degree of heterogeneity within gliomas constitutes a hindrance for the effective isolation and detection of such CTCs. The use of antibodies towards one or few protein surface markers will render the detection fragile to changes in the expression level of the selected marker. On the other hand, targeting several proteins by using an antibody cocktail increases the risk of false positives and high background levels due to healthy cells expressing one or more of the included markers. Hence, a single marker to distinguish a broad repertoire of glioma CTCs from healthy white blood cells (WBCs) is needed. 

Notably, little attention has been given to cancer specific glycosylation patterns on CTCs and strategies for targeting these. Glycosaminoglycans (GAGs) are carbohydrate structures, which are added to proteins, called proteoglycans, as secondary modifications. Chondroitin sulfate (CS) is one type of GAG that is built up by repeated disaccharide units made up of *N*-acetyl-d-galactosamine and d-glucuronic acid units [19]. While the CS backbone structure is simple, an immense heterogeneity is achieved through additional modifications, such as alternate sulfation of component hydroxyl groups [20]. The long structures of repeated disaccharide units are implicated in the regulation of many oncogenic processes and CS up-regulation or modifications have been associated with cancer progression [21]. In the case of glioma, several chondroitin sulfate proteoglycans (CSPGs), including versican and NG2/CSPG4, have been shown to be up-regulated and involved in tumor cell growth, migration, and invasion, as well as in promoting angiogenesis [22,23,24]. 

We have previously shown that the recombinantly expressed VAR2CSA malarial protein (rVAR2) specifically binds a distinct CS structure, termed oncofetal chondroitin sulfate (ofCS), which is present in the placenta and on almost all cancer cells with limited expression in other normal tissues [25]. Although CS is present elsewhere in the vasculature, parasite infected erythrocytes that express VAR2CSA only bind in the placenta [26]. Thus, the protein has been evolutionary refined to specifically bind to ofCS and not to CS present in other organs.

We recently published a CTC isolation method demonstrating the use of rVAR2 protein on magnetic beads for the capture of CTCs from prostate, pancreatic, and hepatic cancer patient blood samples [27]. However, in terms of CTC detection after enrichment, this assay was still dependent on antibody staining using the epithelial marker cytokeratin (CK), thus limiting the applicability to cancers of epithelial origin. In this study, we investigate whether the rVAR2 protein can be applied in both the capture and detection step and thereby broaden the use of our CTC-isolation platform to include circulating glioma cells. We show that rVAR2 binds glioma cells of both adult and pediatric origin. We find that rVAR2 interacts with ofCS on several CSPGs that have shown to be up-regulated in GBM, including CD44, APLP2, CSPG4, PTPRZ1, versican, and syndecan 1. Furthermore, we confirm that rVAR2 binding is retained on a low-grade pediatric glioma cell line (Res259) after incubation with Transforming Growth Factor beta (TGF-β). We validate that the rVAR2-based CTC capture enables capture of rare glioma cells spiked into blood, and show proof-of-concept of using rVAR2 for both the capture and downstream detection of such glioma cells. Importantly, we capture and detect glioma CTCs from glioma patient blood samples. Finally, CTCs from three patient samples are analyzed by whole exome sequencing (WES), which confirms the presence of glioma-associated mutations.

## 2. Materials and Methods

### 2.1. Production of Proteins

The recombinant DBL1-ID2a subunit or the shorter version, ID1-ID2a, of VAR2CSA (rVAR2) was expressed in SHuffle T7 Express Competent *E. coli* (NEB) and purified using affinity chromatography (HisTrap HP, GE Healthcare, Uppsala, Sweden), followed by cation exchange chromatography (HiTrap IMAC SP HP, GE Healthcare). Both constructs included a C-terminal 6x His-tag and V5-tag, as well as an N-terminal SpyTag. For the staining of CTCs, we produced the recombinant ID1-ID2a subunit of VAR2CSA in S2 insect, which encoded an N-terminal twin-strep affinity tag. Protein that was expressed in S2 cells was captured from the supernatant by Streptactin XT chromatography (Iba, GmbH, Germany) and polished by size exclusion (Superdex 200pg, GE). 

Subsequently, purified monomeric proteins were identified by SDS-PAGE. All of the proteins were quality tested by decorin binding in ELISA and by ofCS binding on cancer cells using flow cytometry to ensure specificity.

The SpyCatcher domain was produced in *E. coli* BL21 as a soluble poly-HIS tagged protein, and purified using affinity chromatography (HisTrap, GE Healthcare), followed by anion exchange (HiTrap IMAC Q HP column, GE Healthcare). Purity was determined by SDS page and quality of protein was ensured by testing the capacity to form an isopeptide bond to the Spy-tagged rVAR2 protein. The SpyCatcher was biotinylated using NHS-biotin (Sigma-Aldrich, Steinheim, Germany). NHS-Biotin was dissolved in DMSO and added in 10 molar excess to the SpyCatcher. After a 1-h incubation at room temperature, the biotinylated SpyCatcher was purified using a zeba spin column with a 7 kDa cut off.

### 2.2. Cell Cultures

Janine Erler and Lara Perryman (Biotech Research & Innovation Centre, University of Copenhagen, Denmark) kindly provided the KNS-42, Res259, U87mg, and U118mg cell lines [28]. The U87mg cells were grown in EMEM, Res259 and KNS-42 were grown in DMEM/F12, and U118mg were grown in DMEM GlutaMAX. All culture media were supplemented with 10% fetal bovine serum, penicillin, streptomycin, and l-glutamine (except DMEM). The primary GBM cell, GBM02, was maintained as an in vivo model in NOG mice with ethical approval (2012-15-2934-00636). Tumor xenograft was dissociated using a papain dissociation kit (Worthington). Isolated ex-vivo GBM02 cells were authenticated by STR profiling and grown as neurospheres in Neurobasal media containing B-27 supplement (Gibco), GlutaMax (Gibco), 10 ng/mL EGF, and 10 ng/mL FGF, as described previously [29]. All of the cell lines were passaged at a regular basis and maintained at 5% CO_2_ at 37 °C. 

### 2.3. Flow Cytometry

The cells were grown to 70–80% confluency in appropriate growth media and then harvested in an EDTA detachment solution (Cellstripper^®^, Corning™). 100,000 cancer cells, WBCs from 100 µL RBC lysed blood, or a mixture of both (according to the description in the Results section) were added to each well in a 96 well plate. Cells were incubated with rVAR2 (400 nM–25 nM) for 30 min. at 4 °C. Subsequently, cells were washed twice and then incubated with FITC-labelled anti-V5 antibody (Invitrogen, 1:500) for 30 min. at 4 °C. Finally, the cells were washed twice and analyzed in a LSR-II (BD Biosciences) for staining intensity. Geometric mean fluorescent intensity (MFI) values were normalized to signals that were obtained when only adding the FITC-labelled anti-V5 antibody.

### 2.4. TGF-β Treatment of Res259 Cells

Res259 were seeded in a density of 2400–5200 cells/cm^2^ in DMEM/F12 that was supplemented with 10% FBS in a T25 culture flask. Cells were allowed to attach for 24 h. After this, cells were treated with TGF-β (Cat. no. T7039, Sigma-Aldrich) at a concentration of 20 ng/mL or equal volumes of TGF-β suspension buffer as control (0.2 µm filtered distilled water) for 72 h to induce the mesenchymal transition. Transition was confirmed by changes in the expression of mesenchymal protein markers using western blot as well as changes in morphology.

For western blot analysis, the cells were lysed with EBC lysis buffer containing PhosSTOP (Sigma-Aldrich) and cOmplete EDTA-free Protease Inhibitor Cocktail (Roche) for 30 min. Protein extract was balanced using Bradford assay. An equal amount of protein lysates were loaded onto a NuPAGE 4–12% Bis-Tris gel (ThermoFisher Scientific), after which the samples were transferred to a nitrocellulose membrane (Biorad). Membranes were blocked in 5% skimmed milk powder in TBS-T. Anti-GAPDH (14C10) antibody (Cell Signaling, 1:1000), and anti-β-catenin (1:500), anti-*N*-cadherin (1:500), and anti-vimentin (1:1000) primary antibodies from the EMT Antibody Sampler Kit (Cell Signaling) were added to the membranes in TBST-T supplemented with 2% skimmed milk powder and incubated overnight at 4 °C. Following three washes in TBS-T, the membranes were incubated with HRP-linked goat anti-rabbit IgG (Cat. no. P0448, Dako, 1:2000) for 1 h at room temperature and the reactivity was detected using LumiGlo Reserve Chemiluminescent Substrate (KPL). Uncropped images of the membranes can be seen in Figure S6.

For fluorescent visualization of changes in morphology, the cells were grown on glass slides and fixed in 4% paraformaldehyde (PFA), washed three times in PBS, blocked with 1% BSA in PBS, and stained with Alexa Fluor^®^ 594 Phalloidin (ThermoFisher, 1:40) for 20 min. at room temperature. Cells were subsequently stained with DAPI (Life Technologies) and mounted using FluorSave Reagent (Merck Millipore, Darmstadt, Germany). Staining was analyzed using Nikon TE2000-E C1 confocal microscope with 60× oil immersion objective lens (DIC).

For flow cytometry-based analysis of cells before and after induction with TGF-β, applying the same procedure as in “2.3. Flow Cytometry”.

### 2.5. Immunoprecipitation and Proteomics

Membrane proteins were extracted by lysing the cells with EBC lysis buffer supplemented with a protease inhibitor cocktail (Roche). Biotinylated rVAR2 was added to the lysate and the mix was incubated overnight at 4 °C. The rVAR2 and bound protein was pulled down on streptavidin-coated dynabeads (MyOne C1, Invitrogen).

The pulled down lysate was dissolved in non-reducing LDS loading buffer (Invitrogen). The protein was reduced in 1 mM DTT and alkylated with 5.5 mM iodoacetamide. The samples were then run 1 cm into Bis-Tris gels and stained with coomasie blue. The protein was cut out, washed, and in-gel digested with trypsin. The resulting peptides were captured and washed using a C18 resin stage-tipping [30]. The peptides were sequenced using a Phusion Orbitrap Mass Spectrometer. Sample analysis and hit verification was performed using the MaxQuant software. All of the samples were verified against the control samples of cell lysates without rVAR2.

### 2.6. Proximity Ligation Assay (PLA)

The PLA protocol was run according to the manufacturer’s instructions (Sigma-Aldrich). U87mg, U118mg, and KNS-42 cells were seeded on laminin-coated coverslips and fixed in 4% PFA. Unspecific binding of antibodies was minimized by incubating with a blocking solution with 1% BSA and 5% FBS in PBS for 1 h at room temperature. The samples were incubated with primary antibodies together with rVAR2 or rDBL4 over night at 4 °C in the following concentrations: rVAR2 (50 nM), rDBL4 (50 nM), anti-NRP1 (Cat. no. ab81321, 1:250), anti-NRP2 (Cat. no. sc-13117, 1:50), anti-PTPRZ1 (Cat. no. HPA015103, 1:61), anti-VCAN (Cat. no. HPA004726, 1:50), anti-CSPG4 (Cat. no. ab20156, 1:200), anti-DCN (Cat. no. PA5-27370, 1:100), anti-CD44 (Cat. no. BBA10, 1:200), anti-SDC1 (Cat. no. ab34164, 1:50), and anti-SDC4 antibody (Cat. no. HPA005716, 1:80). Between incubations, the cells were washed in Wash Buffer A (DUO82049, Sigma-Aldrich). An anti-V5 antibody (Invitrogen, 1:500) was used to detect rVAR2. The cells were then stained with Duolink^®^ In Situ PLA^®^ Probe Anti-Mouse MINUS (DUO92004) and Duolink^®^ In Situ PLA^®^ Probe Anti-Rabbit PLUS (DUO92002) diluted in Antibody Diluent (DUO82008). The cells were then treated with the ligation solution, followed by incubation with the amplification solution, which were provided with the kit Duolink^®^ In Situ Detection Reagents Orange (DUO92007). The cells were washed with Wash Buffer B (DUO82048). The slides were mounted using Duolink^®^ In Situ Mounting Medium with DAPI (DUO82040), and the results were then analyzed under a Nikon TE2000-E C1 confocal microscope with a 60× oil objective. A total of 75–100 cells were imaged per sample. The images were analyzed using the BlobFinder software (version 3.2.). Negative controls using the recombinant DBL4 domain of VAR2CSA are found in Figure S1.

### 2.7. Immunocytochemistry of Cancer Cells Mixed with White Blood Cells (WBCs)

Two mL blood from a healthy donor was drawn in a LBgard^®^ vacutainer (BioMatrica). The blood sample was diluted 10 times in Red Blood Cell (RBC) lysis buffer resulting in a final concentration of 0.155M ammonium chloride, 0.01M potassium hydrogen carbonate and 0.1 mM EDTA, and incubated for 13 min. After centrifugation at 400× *g* for 8 min. the pelleted cells were resuspended in 2 mL Dulbecco’s PBS (without Ca^2+^ and Mg^2+^) supplemented with 2% Fetal Bovine Serum (FBS) (Gibco) and transferred to eppendorf tubes in aliquots of 0.5 mL. Res529, KNS-42 and U87mg were detached using 1 mL CellStripper (Corning™) and resuspended in their respective media. Approximately 2000 cells were added to each aliquot of WBCs. The samples were washed once, prior to incubation with a cocktail of 200 nM rVAR2, CF488-labelled anti-V5 (Cat.no. 20440, Biotium, 1:150), PE-labelled anti-CD45 [5B1] (Cat.no. 170-078-081, MACS Miltenyi Biotec, 1:40) and PE-labelled anti-CD66b [REA306] antibodies (Cat.no. 130-104-396, MACS Miltenyi BioTec, 1:40) in Dulbecco’s PBS with 2% FBS for 30 min. at 4 °C. Finally, the cells were fixed with 4% PFA, stained with DAPI (Cat. no. D1306, Life Technologies) and mounted using Faramount Aquous Mounting Media (Dako). The slides were imaged using the 10× objective of Cytation 3 Cell Imaging Multi-Mode Reader (BioTek, Europe).

### 2.8. Preparation of rVAR2-Coated Beads

The Spy-tagged DBL1-ID2a or ID1-ID2a (rVAR2) was mixed with the biotinylated SpyCatcher in a 1.2:1 ratio and then incubated at room temperature for 1 h. After this step, the biotinylated rVAR2 protein was incubated with CELLection™ Biotin Binder Dynabeads^®^ (4.5 µm) at room temperature for at least 30 min. resulting in rVAR2-coated beads (0.43 µg biotinylated protein per µL bead suspension). The remaining protein or antibody was removed by carefully washing the beads in Pierce™ Protein-Free (PBS) blocking buffer (Cat. no. 37572, ThermoFisher) three times, each time using a neodymium magnet (10 × 12 mm) for dragging beads into a pellet.

### 2.9. Spike-In Experiments

Prior to the spike-in experiments, the cancer cells were harvested with CellStripper (Corning™) or TrypLe (Cat. no. 12604013, Gibco) (only used for GBM02 cells) and resuspended in culture medium. For spike-in experiments measuring the efficiency of recovery, cancer cells were prestained using CellTracker™ Green CMFDA Dye (Cat. no. C7025, ThermoFisher), according to manufacturer’s protocol for cells in suspension. Following staining, the cells were resuspended in complete growth media and incubated for 30 min. under normal growth conditions in order to recover.

Cell concentration was measured by manually counting the number of viable cells in a 1:1 mixture with Trypan Blue solution (Sigma-Aldrich). Subsequently, the cells were diluted to 10,000 cells/mL in Dulbecco’s PBS and the desired number of cells were spiked into 3 mL blood. Triplicates of the spike-in volume (e.g., 10 μL for 100 cells) were placed on a glass slide and cells were manually counted under a light microscope (10× objective) in order to confirm the exact number of cells spiked into the blood. The average of the cell counts was used when calculating the percentage of recovery. Each of the spike-in experiments were repeated at least twice with 2–4 replicates per test.

When spiking in low cell numbers (5–10 cells), serial dilutions were made using cell culture media. The cell suspension was transferred to a 96-well plate and counted under a light microscope (10× objective) to ensure precise cell count before spike-in. Finally, cells were directly added from the well to the 3 mL blood sample.

After adding the cancer cells to the blood, the samples were immediately processed, as described in Section 2.11.

### 2.10. Patient Samples

Up to 9 mL blood samples from glioma patients were collected under ethical approval (journal no. H-3-2009-136). Informed written consent was obtained for all of the enrolled subjects. Blood was received in K2 EDTA-tubes and processed within 2 h of collection.

### 2.11. CTC Isolation from Blood

Three mL blood samples were lysed in 27 mL RBC lysis buffer reaching a final concentration of 0.155 M ammonium chloride, 0.01 M potassium hydrogen carbonate, and 0.1 mM EDTA for 13 min. After centrifugation at 400× *g* for 8 min., the cell pellet was gently washed in Dulbecco’s PBS. The centrifugation step was repeated, and finally the cells were resuspended in 0.6 mL Pierce™ Protein-Free (PF) PBS blocking buffer (Cat. no. 37572, ThermoFisher) and then transferred to a low retention microcentrifuge tube (Fisherbrand). Under these conditions, the cells were incubated with ~1.8 million rVAR2-coated magnetic beads at 4 °C for 20 min. A neodymium cylinder magnet was used to drag cells bound to beads towards the side of the tube, enabling removal of supernatant. Cells were then fixed in 4% PFA for 5 min. and resuspended in Pierce™ Protein-Free (PBS) blocking buffer diluted 1:10 in Dulbecco’s PBS.

### 2.12. CTC Staining and Enumeration

For spike-in experiments where the cells were prestained with CellTracker Green, cells were stained with DAPI (Cat. no. D1306, Life Technologies) diluted in Dulbecco’s PBS with 0.5% BSA and 2 mM EDTA for 5 min. at room temperature. Following one wash, the bead-bound cells were added to a SensoPlate™ (24-well, glass bottom) (Cat. no. 662892, Greiner Bio-One). Excess liquid was removed by holding the bead-bound cells in place with a magnet underneath the plate, and the samples were mounted using Faramount Aqueous Mounting Media (DAKO). The entire well was scanned for DAPI and CellTracker signal using the Cytation™ 3 Cell Imaging Multi-Mode Reader and manually enumerated using the Gen5 software (BioTek).

For patient samples and spike-in experiments with non-prestained cells, the isolated cells were briefly blocked in Dulbecco’s PBS containing 2% FBS, followed by incubation in a non-protein based blocking solution. The cells were then incubated with a mixture of 200 nM fluorophore-conjugated (Oregon Green^®^ 488) rVAR2 and PE-labelled anti-CD45 [5B1] (Cat. no. 170-078-081, MACS Miltenyi Biotec, 1:10) and PE-labelled anti-CD66b [REA306] (Cat. no. 130-104-396, MACS Miltenyi BioTec, 1:20) antibodies for 30 min. at room temperature and then washed once in PBS with 2% FBS to remove excess staining reagents. Finally, the cells were DAPI-stained and mounted on a Sensoplate. Duplicates of 3 mL patient blood were imaged using the Cytation™ 3 Cell Imaging Multi-Mode Reader. Additional 1–2 mL blood was processed according to the above description, except that the exclusion marker that was used in this setup was APC-labelled anti-CD45 [HI30] antibody (Cat. no. 17-0459-42, ThermoFisher), and analyzed using the CellCelector™ (Automated Lab Solutions).

For spike-in experiments testing anti-CSPG4 antibody as a staining reagent after rVAR2 bead pull-down, cells were blocked in 10% Normal Donkey Serum (NDS), 0.5% BSA, and 2 mM EDTA in Dulbecco’s PBS for 10 min. After this, the cells were incubated with anti-CSPG4 (Cat. no. ab20156, Abcam, 1:100) antibody diluted in Dulbecco’s PBS with 1% NDS, 0.5% BSA, and 2 mM EDTA for 30 min. at room temperature. The cells were washed once in Dulbecco’s PBS containing 0.5% BSA and 2 mM EDTA and then incubated with anti-mouse IgG-FITC (Vector, Cat. no. FI-2000, 1:400) for 30 min. at room temperature. Finally, the cells were washed in Dulbecco’s PBS containing 0.5% BSA and 2 mM EDTA, DAPI stained, and mounted on a Sensoplate.

### 2.13. Classification and Enumeration of rVAR2-Stained Cancer Cells or CTCs

The samples were scanned on a 10× objective using the Cytation™ 3 Cell Imaging Multi-Mode Reader and manually enumerated using the Gen5 software (BioTek). Putative CTCs were defined as DAPI+, CD45/CD66b−, and rVAR2+. The signal to noise ratios were adjusted according to the fluoresence of the CELLection Biotin Binder beads, such that a staining was only regarded as positive if the intensity was above the fluorescence from the beads. Furthermore, all the cells with a DAPI area below 4 μm were excluded from enumeration.

### 2.14. Single Cell Picking and Whole Genome Amplification

Cell samples were resuspended in 200 µL PBS and then loaded onto a CellCelector™ magnetic slide (Automated Lab Solutions) to align and preserve the localization of the magnetic beads and cells during scanning. Employing the CellCelector™, the samples were then screened for coinciding Origon Green^®^ 488 and DAPI signals as well as absent APC fluorescent signals, thereby detecting potential CTCs. Single cells were picked by the CellCelector™ and then pooled into PCR tubes containing 5 µL lysis buffer and enzyme from the MALBAC^®^ Single Cell WGA Kit (Yikon Genomics, Cat. no. EK100101210). Each tube was prepared with 10–20 cells. Whole genome amplification (WGA) was performed on picked CTCs or WBC controls, according to manufacturer’s instructions. The quality of the WGA products was verified by agarose gel electrophoresis and concentrations were measured by Qubit™ dsDNA BR Assay Kit (Cat. no. Q32850, Thermofisher Scientific).

### 2.15. Whole Exome Sequencing

Whole exome sequencing on whole genome amplified DNA from isolated CTCs and patient-matched WBCs was performed as previously described [31]. The Exome sequencing data was aligned against the human reference genome (hg19/GRCh37) using bwa mem 0.7.15 and somatic SNVs and small indels were called using Mutect2 according to the GATK best practices for somatic short variant discovery using GATK 4.1.0.0. Variants outside a selected glioblastoma-related target region containing 95 candidate genes were excluded from the call set. Mutect2 was provided with data from picked, patient-specific WBCs as a matched reference sample to reduce the amount of germline variants in the call set, hence obtaining a list of somatic mutations only for the CTC samples. The most relevant mutations (described in somatic mutation databases or being frameshift/stop-gain) were further manually inspected by looking at aligned reads sequences. The Integrative Genomics Viewer (Broad Institute, UK) was used for the visualization of variants (Figure S4) [32].

## 3. Results

### 3.1. rVAR2 Binds to ofCS on Glioma Cells

**Figure 1:**
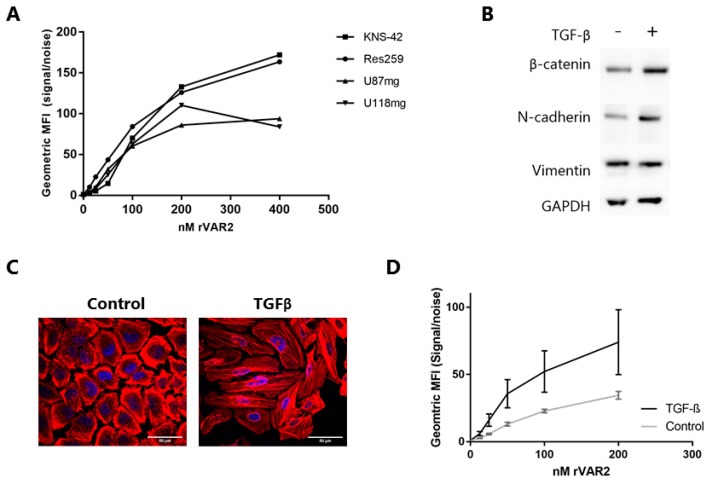
Recombinant malaria VAR2CSA protein (rVAR2) binds to glioma cells and the binding is unaffected by phenotypic changes. (**A**) rVAR2 binding to the glioma cell lines KNS-42, Res259, U87mg and U118mg was measured by flow cytometry using a FITC-conjugated anti-V5 antibody. Geometric Mean Fluorescence Intensity (MFI) was measured after incubation of cells with various rVAR2 concentrations. Results are displayed as signal/noise ratio. Figure represents data from one experiment, replicates are found in Figure S2. (**B**) Western blot of Res259 cell lysates after 72 h incubation with Transforming Growth Factor-beta (TGF-β) or buffer control. Membranes were incubated with anti-β-catenin, anti-*N*-cadherin, anti-Vimentin or anti-GAPDH antibodies and detected by anti-rabbit HRP antibody. (**C**) Representative images of fixed Res259 cells after 72 h incubation with TGF-β or buffer control. Cells were stained with phalloidin to stain F-actin (red) and DAPI to stain nuclei (blue). Scale bars, 50 µm. (**D**) rVAR2 binding to Res259 incubated with TGF-β or buffer control for 72 h measured by flow cytometry (*p* < 0.001, generalized least squares regression model). Geometric MFI was measured after incubation of cells with various rVAR2 concentrations and a FITC-conjugated anti-V5 antibody. Results are displayed as signal/noise ratio. Bars show standard deviation (n = 3).

We have previously shown that the rVAR2 protein interacts with ofCS present on cancer cell lines representing almost all known cancers [25,33,34]. We tested rVAR2 binding to a panel of cell lines, including low-grade (WHO grade II) diffuse glioma (Res259) as well as high-grade (WHO grade IV) GBM (U87mg, KNS-42, and U118mg), to test for the presence of ofCS in glioma. All of the cell lines were positive for rVAR2 binding by flow cytometry, indicating that the glioma lines expressed ofCS (Figure 1A and Figure S2).

Cell cultures poorly represent the phenotypic plasticity of cancer cells in vivo, where the tumor cells continuously respond to signals from the microenvironment. TGF-β, for instance, is known to enhance the migratory and invasive capability of glioma cells, most likely by pushing these cells towards a more mesenchymal phenotype [35,36]. Therefore, we evaluated whether TGF-β exposure of the low-grade Res259 glioma cell line affected the expression of ofCS, as measured by rVAR2 binding. It should be noted that, although glial cells originate from ectodermal tissue, these cells exhibit a more mesenchymal appearance, such as the expression of vimentin [28]. Thus, the transition is measured as an increased expression of mesenchymal markers, rather than a down-regulation of epithelial markers [36]. After incubation with TGF-β, the Res259 cells showed increased expression of the mesenchymal markers β-catenin and *N*-cadherin in accordance with a transition towards a more mesenchymal state (Figure 1B). This was accompanied by a clear change in morphology as cells tended to become more elongated, which confirms the occurrence of a transition [36,37] (Figure 1C). Importantly, when testing for rVAR2 binding to Res259 cells in flow cytometry before and after incubation with TGF-β, rVAR2 binding was not reduced (Figure 1D).

### 3.2. rVAR2 Captures Glioma Cancer Cells Spiked Into Blood

**Figure 2:**
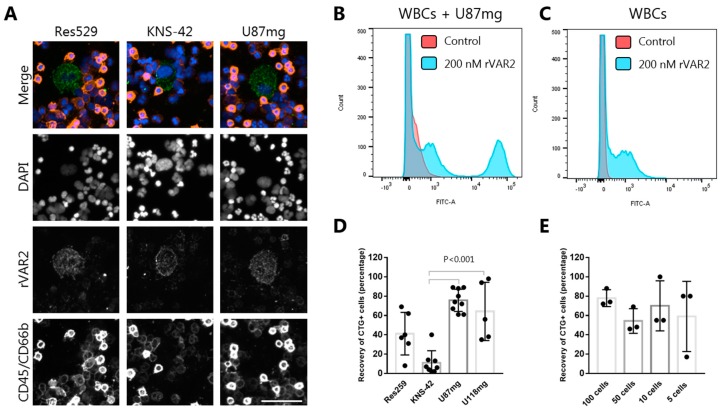
rVAR2 specifically binds to glioma cells and enables their retrieval from blood. (**A**) rVAR2 stains glioma cell lines in a background of white blood cells (WBCs). Res529 (left), KNS-42 (middle) and U87mg (right) cells were mixed with WBCs and stained with V5-tagged rVAR2 in combination with a CF488-conjugated anti-V5 antibody (green), PE-conjugated anti-CD45 and anti-CD66b antibodies (red), and DAPI (blue). Scale bar, 50 µm. (**B**) Flow cytometry analysis showing WBCs (100 µL RBC lysed blood) mixed with U87mg (50,000) cells and detected with either 200 nM rVAR2 and a FITC-conjugated anti-V5 antibody or with a FITC-conjugated anti-V5 antibody alone (control). (**C**) Same as in (**B**) but with no U87mg cells added. (**D**) Recovery of CellTracker Green-stained glioma cells from blood. 100 cells were spiked into 3 mL blood and recovered using rVAR2-coupled beads. Cells were stained with DAPI and scanned on the Cytation 3 Imager. Each dot represents the percentage of recovered cells from one sample. Bars represent mean recoveries and error bars show +/− standard deviation (n ≥ 2) (one-way ANOVA with Bonferroni correction). (**E**) Recovery of CellTracker Green-stained U87mg cells from blood. The indicated number of U87mg cells was spiked into 3 mL blood and captured using rVAR2-coupled beads. Enumeration of cells and data presentation were done as in (**D**).

To examine whether rVAR2 can be used for targeting circulating glioma cells, it is pivotal to ensure the specificity of rVAR2 binding to glioma cells in a background of normal white blood cells (WBCs). Therefore, WBCs from 0.5 mL blood were mixed with 2000 U87mg, Res259, or KNS-42 cells and incubated with rVAR2 and a CF488-labeled anti-V5 antibody. rVAR2 binding showed a clear and specific membrane staining of all three cancer cell lines with minimal staining of the surrounding WBCs (Figure 2A). This was further tested by flow cytometry analysis showing specific binding to U87mg cells when mixed with WBCs (Figure 2B,C).

To examine whether rVAR2 binding to glioma cell lines could support magnetic capture and isolation of these cells from whole blood, 100 glioma cells were prestained using a CellTracker™ Green CMFDA Dye and spiked into a 3 mL blood sample from a healthy individual. This strategy allowed us to directly assess the recovery of spiked cells independent of downstream staining and detection biases. After lysis of the erythrocytes, cancer cells were isolated using rVAR2-coated magnetic beads (see Method section for details) [27]. By this procedure, we achieved an average recovery of 76%, 41%, 11%, and 64% for U87mg, Res259, KNS-42, and U118mg cells, respectively (Figure 2D). Furthermore, we spiked 3 mL blood samples with 100, 50, 10, or 5 U87mg cells to assess the sensitivity of the assay (Figure 2E). The average recovery ranged between 54–75% with no obvious association to the number of cells spiked into the blood (*p* = 0.31, one-way ANOVA). Although the recovery varied between the different cell lines, this data confirms that rVAR2 can be used as a capture molecule for the isolation of various glioma cell types.

### 3.3. rVAR2 Interacts with Several GBM-Associated Proteoglycans

**Figure 3:**
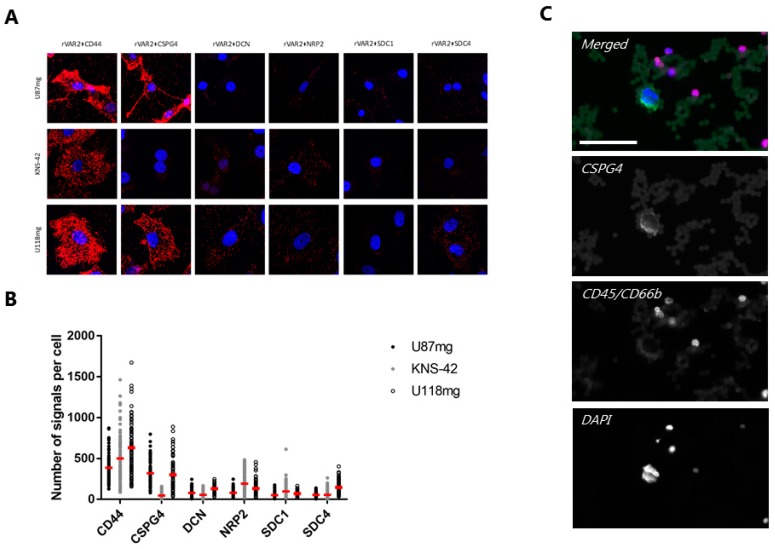
Evaluation of protein pull-down hits from mass spectrometry by proximity ligation assay (PLA). (**A**) Representative images of PLA assays on U87mg, KNS-42, and U118mg cells showing co-localization between rVAR2 and a panel of CSPGs as red dots. Cells were counterstained with DAPI (blue) and analyzed by confocal microscopy. All of the images are shown in same magnification using a 60× objective. (**B**) Quantification of the PLA co-localization signals between rVAR2 and each of the CSPGs analyzed. Data is shown as the number of signals per cell. Red bars represent the mean number of signals per cell. (**C**) Representative image showing specific CSPG4 staining (green) of an rVAR2-captured U87mg cell in a background of WBCs stained for CD45 and CD66b (both red). Cells were stained with DAPI (blue) and visualized on the Cytation 3 Imager with a 20× objective. Scale bar, 50 µm.

We have previously described that single cancer cells simultaneously display the ofCS modification on several proteoglycans [33]. We analyzed rVAR2-based pull-down of lysates from KNS-42, U118mg, and U87mg cell lines to investigate the proteoglycan display on glioma cells. The mass spectrometry results showed the pull-down of multiple key cancer-related proteoglycans (Table 1). Among the hits were several chondroitin sulfate proteoglycans (CSPGs) that have been described for GBM, such as CSPG4, CD44, APLP2, and SDC1 [38,39,40,41]. To validate these findings, we studied the co-localization of ofCS and selected protein cores from the pull-down proteomic list by proximity ligation assay (PLA) (Figure 3A). Indeed, compared to other proteoglycans rVAR2 binding and CD44 showed a strong co-localization on each evaluated glioma cell line (*p* < 0.001, one-way ANOVA) (Figure 3B). Despite the high PLA signal, CD44 was not further examined as a potential CTC marker, as anti-CD44 antibodies are also found to target a subset of healthy WBCs [42]. Similarly, ofCS and CSPG4 were clearly co-localizing on U87mg and U188mg cells (*p* < 0.001, one-way ANOVA) (Figure 3B). Interestingly, anti-CSPG4 antibodies are already being used for the capture and detection of circulating melanoma cells [43,44,45,46]. Since CSPG4 is also an emerging target for GBM CAR-T immunotherapy, we examined whether CSPG4 is still accessible for antibody staining after rVAR2-based capture [47,48]. Indeed, the captured U87mg cells showed clear and specific CSPG4 staining in a background of WBCs (Figure 3C). Hence, the capture of glioma CTC might be useful for predicting response to anti-CSPG4 CAR-T therapy.

### 3.4. rVAR2 Detects Cancer Cells Spiked Into Blood Samples

**Figure 4:**
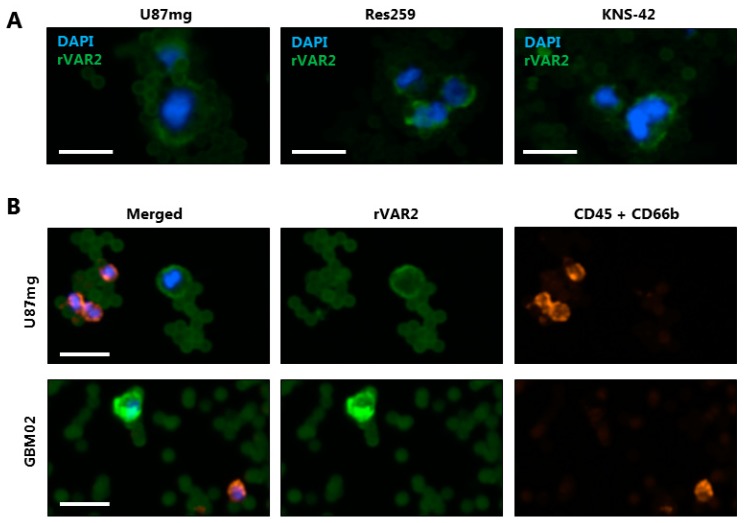
Using rVAR2 to stain glioma cells after capture with rVAR2-coupled beads. (**A**) Glioma cell lines (U87mg, Res259, and KNS-42) were incubated with rVAR2-coupled beads and stained with a fluorophore-conjugated rVAR2 (green) and DAPI (blue). Representative images were obtained using the Cytation 3 Imager with a 10× objective. Scale bars, 20 µm. (**B**) U87mg cells and GBM02 cells were spiked into 3 mL blood, retrieved using rVAR2-coupled beads, and stained using fluorescent rVAR2 (green), anti-CD45/CD66b antibodies (red) and DAPI (blue). Scale bars, 20 µm.

All of the CTC capture protocols will, even in the best of circumstances, capture some normal WBCs along with the CTCs. A major challenge associated with the analysis of glioma CTCs is how to validate which captured cells are indeed cancer cells and not WBCs. With regard to carcinoma-derived CTCs the most widely used markers are EpCAM or CK. While gliomas are EpCAM negative, the results regarding CK-positivity are less consistent [12,49,50,51]. Thus, these markers are not optimal for the detection of glioma CTCs. Therefore, we established a platform where rVAR2-coupled beads were used for capturing, while a fluorophore-conjugated rVAR2 was used for microscopic detection of the captured cells. U87mg, Res259, and KNS-42 cells all showed rVAR2 staining after capture with rVAR2-coupled beads (Figure 4A and Figure S3). However, it was noticed that rVAR2-staining of U87mg after magnetic capture was somewhat reduced compared to the Res259 and KNS-42 cells. Next, we applied the same workflow to U87mg cells that were spiked into 3 mL healthy donor blood in order to mimic patient blood samples. The rVAR2 staining enabled detection of U87mg cells and their separation from CD45- and/or CD66b-positive WBCs (Figure 4B). The strategy was also effective with cells from a primary glioblastoma cell culture, GBM02 (Figure 4B).

### 3.5. rVAR2 Captures and Detects CTCs in Glioma Patient Blood Samples

**Figure 5:**
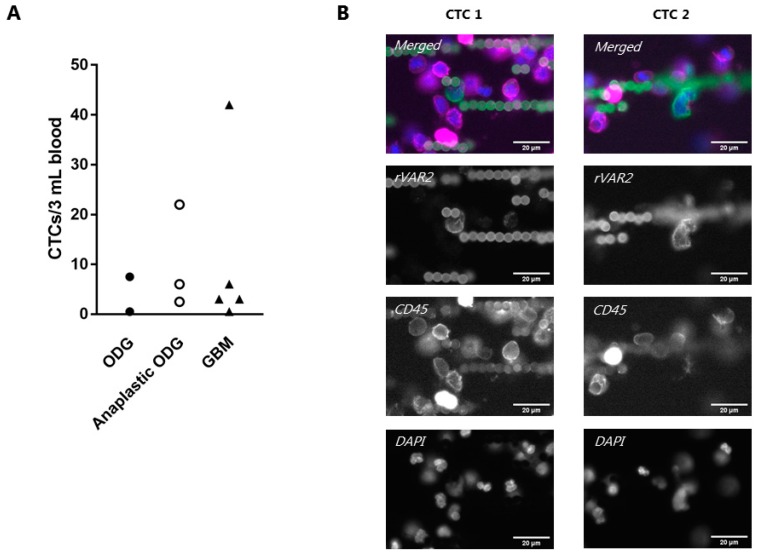
rVAR2 enables capture and detection of glioma circulating tumor cells (CTCs) from patient blood samples. (**A**) Average CTC count in blood samples from ten glioma patients. 3 mL blood samples were processed by using rVAR2-coupled beads followed by staining using a mixture of fluorophore-conjugated rVAR2 (green), anti-CD45/CD66b antibodies (red) and DAPI (blue). CTCs were defined as rVAR2+, CD45/CD66b−, DAPI+ cells. Each dot represents the average number of detected CTCs per 3 mL patient blood sample. The x-axis shows whether the patient was diagnosed with GBM, Anaplastic Oligodendroglioma (ODG), or Oligodendroglioma (ODG). (**B**) Representative images of identified CTCs from a patient diagnosed with GBM. The sample was stained with flurophore-conjugated rVAR2 (green), anti-CD45 antibody (magenta), and DAPI (blue). Images were obtained using the CellCelector™ (ALS) with a 40× objective. Scale bars, 20 µm.

We tested for the presence of CTCs in blood samples from glioma patients using the combined rVAR2 capture and detection protocol. Duplicates of 3 mL blood samples from 10 glioma patients, suffering from oligodendroglioma (grade II), anaplastaic oligodendroglioma (grade III), or GBM (grade IV), were processed and visualized for enumeration. CTCs were manually enumerated as rVAR2+, CD45/CD66b−, and DAPI+ cells. The range of identified CTCs per 3 mL blood was 0.5–42 (Figure 5A). There was no obvious correlation between grade or type of diagnosis and CTC number. Interestingly, one patient who had progressed from an initial diagnosis of oligodendroglioma (grade II) to anaplastic oligodendroglioma (grade III) within a time span of 15 years had a relatively high number of CTCs (22 CTCs per 3 mL blood). In a patient with the reverse clinical history regressing from an initial diagnosis of anaplastic oligodendroglioma (grade III) to eight years later having oligodendroglioma (grade II) we detected an average of only 0.5 CTCs per 3 mL. Representative images of rVAR2+ cells from one of the GBM patients are shown (Figure 5B) and the full list of detected CTCs is found in Figure S5.

### 3.6. Captured Glioma CTCs Show Cancer-Indicative Mutations

**Figure 6:**
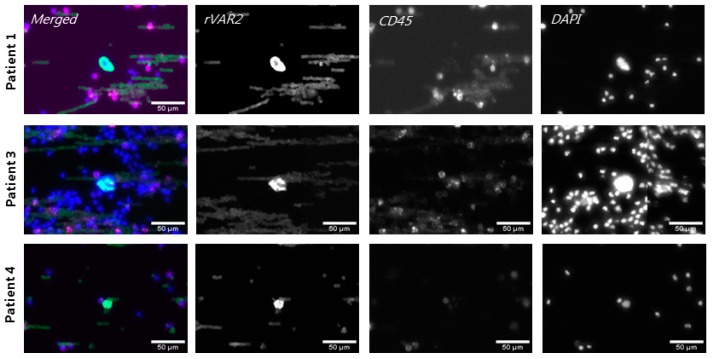
Identification of CTCs for whole exome sequencing (WES). Representative images of identified CTCs from three patients. Cells were stained with fluorophore-conjugated rVAR2 (green), anti-CD45 antibody (magenta) and DAPI (blue) and classified as CTCs if rVAR2+, CD45−, and DAPI+. Images were obtained using the CellCelector™ (ALS) with a 10× objective. Scale bars, 50 µm.

To confirm that the VAR2+, CD45− cells detected in the patient blood samples were indeed glioma-derived CTCs, we performed targeted whole exome sequencing (WES) searching for glioma relevant mutations. For three patient samples we single cell picked rVAR2+, CD45− cells, and patient-matched WBCs as germline controls using an ALS CellCelector™. For each patient, 2–4 CTCs were pooled into one sample and whole genome amplification (WGA) was performed (Figure 6). However, since WBCs were located close to some of the selected CTCs, the cell picking procedure resulted in samples containing CTCs together with some WBCs (Table 2). The WGA product was then used for WES. The WES results were filtered to only include glioma relevant mutations and each hit was visually confirmed by evaluating the IGV screen shots (Figure S4). Indeed, we identified genes with cancer-indicative mutations in all CTC samples: RB1, TP53/EPM2AIP1, and TP53/ALK for patient 1, 3, and 4, respectively (Table 2). Thus, the molecular profiling supports the tumor origin of the picked patient-derived CTCs.

## 4. Discussion

The isolation and characterization of glioma CTCs have proven to be challenging, especially concerning the detection and validation of the tumor origin of the isolated cells. To date, only a few studies have shown the presence of circulating glioma cells utilizing either a single antibody marker or an antibody mixture for CTC detection [13,14]. Here, we present a novel strategy for glioma CTC capture and detection based on targeting the unique cancer-specific glycosaminoglycan structure ofCS. We show that rVAR2, which binds ofCS with high affinity, specifically targets a panel of glioma cell lines in a background of white blood cells (WBCs). Furthermore, we show that rVAR2 can be used for the capture of glioma cells that are spiked into blood by coupling the protein to magnetic beads. In addition, the staining of glioma cells with a fluorophore-conjugated rVAR2 after magnetic pull-down facilitates their detection and separation from WBCs. This workflow was applied to blood samples that were derived from ten glioma patients and established proof-of-concept for identification of glioma CTCs. In three of the patients, potential CTCs were picked and molecular analysis supported their tumor origin.

Flow cytometry analysis showed rVAR2 binding to all tested glioma cell lines. Interestingly, the cell lines showed varying maximum intensity at saturation indicating different levels of ofCS display. In all of the experiments, U87mg showed the lowest level of rVAR2-binding. This is interesting, since U87mg cells had the highest recovery when spiked into blood and isolated with rVAR2-coupled beads. This could indicate that efficiency of recovery, not only depends on the level of target expression but is also influenced by other factors, such as the capability of a given cell line to survive through the experimental workflow. When using spike-in of cancer cells in healthy donor blood, the experimental procedure, among others, includes detachment from the culture plate and exposure to the various components of a foreign immune system when spiked into blood. Patient-derived CTCs do indeed experience dramatic changes in physical conditions upon entering the circulation, such as shear stress forces and the loss of cell-cell or cell-matrix attachment. However, it could be debated how comparable this sequence of events is to the in vitro spike-in models, and thus how well cell line spike-in samples reflect the phenotypes of CTCs in patient-derived liquid biopsies.

Elevated levels of TGF-β in the tumor microenvironment and a mesenchymal phenotype of the glioma cells have independently been shown to be associated with a poor prognosis in glioma patients [36]. TGF-β is known to induce increased motility and invasive behavior, which underlines a potential link between cellular plasticity and intravasation of cancer cells into the bloodstream [52]. Notably, the expression of surface markers might be altered during such phenotypic changes, and this process should be taken into consideration when deciding on a capture and detection reagent for CTC capture. In line with other studies, we have previously shown that an EMT-like process can be induced in U87mg cells by incubating with TGF-β for 72 h [27]. Importantly, we also confirmed that rVAR2 binding to U87mg cells was maintained after the transition. However, several studies indicate that the EMT-like processes also play a role in the progression from low-grade to high-grade gliomas [53,54]. Here, we confirmed that ofCS display is retained when the Res259 low-grade glioma cell line is pushed towards a more mesenchymal morphology and protein expression pattern by incubation with TGF-β. This strengthens the potential of using ofCS as a target, not only for the capture, but also for the detection of glioma CTCs.

When considering previously published data showing that ofCS is presented by nearly all cancer cells of epithelial, mesenchymal, and hematopoietic origin, the use of rVAR2 staining reagent for CTC detection would be beneficial over traditional single-surface markers [25,27,33,34]. Here, we show that captured glioma CTCs can be identified by an rVAR2 stain. However, one should be cautious when using the same target for both capture and detection, as the general assumption is that the use of two independent markers would lead to a better exclusion of false positive hits. Furthermore, as CS is a common GAG that is displayed on all cell types, including WBCs, an extremely high degree of ofCS-specificity is needed to successfully capture and distinguish CTCs from WBCs. Interestingly, the naturally occurring VAR2CSA that is expressed by malaria-infected erythrocytes serves exactly this purpose, since binding to normal WBCs would result in parasite clearance [26]. However, the use of exclusion markers is highly important to exclude potential false positives. In the workflow presented here, we included CD45 and CD66b as exclusions markers to identify and reject a broad repertoire of WBCs. The optimized and combined rVAR2 capture and detection workflow enabled us to isolate and detect circulating glioma cells in glioma patients. In this very limited dataset, the number of CTCs detected did not correlate with type of diagnosis or WHO grade.

A potential clinical application of rVAR2-based CTC detection could be patient stratification based on the expression of therapeutically relevant CSPGs on CTC subsets. In this study, we sought to identify ofCS-modified proteoglycans in glioma by using rVAR2-based protein pull-down of lysates from KNS-42, U118mg, and U87mg cell lines. Indeed, the subsequent proteomics analysis showed the pull-down of multiple cancer-related proteoglycans with key roles in the pathogenesis of glioma. Unlike our previous study showing syndecan 1 to be the main VAR2CSA receptor in the placental syncytium [55], we found several interesting hits on the glioma cell lines, including syndicans, glypicans, neuropilins, decorin, versican, CSPG4, and PTPRZ1. The two last mentioned are currently being explored as potential anti-cancer targets in GBM [56,57,58]. In this study, we tested the use of anti-CSPG4 antibodies for staining and detection of cancer cells after rVAR2 capture, which could potentially be of future interest in the monitoring of anti-CSPG4 CAR-T therapies. Finally, CD44 was identified as a hit on all of the glioma cell lines. High CD44 expression is common in GBM and is used to identify GBM with particular poor survival chance [59,60]. Along this line, CD44 is expressed by GBM cancer stem cells, which promotes aggressive GBM growth [61]. Thus, adding a CD44-stain to rVAR2 captured CTCs could provide additional information regarding predicted outcome if sufficient exclusion markers are included. Another interesting application of the captured CTCs could be to culture and further characterize the CTCs in terms of responsiveness to relevant treatments. Liu et al. has shown proof-of-concept by culturing CTCs that are captured from a mouse GBM model [62]. To our knowledge, no one has to date been able to culture the sparse number of CTCs found in glioma patient blood samples.

We picked CTCs and performed WGA followed by WES against a panel of known glioma mutations to confirm that the detected rVAR2+, CD45−, and DAPI+ cells were actual CTCs derived from the brain tumors. Patient-matched WBCs were used as germline subtractions. Patient I, which was diagnosed with anaplastic oligodendroglioma, had CTCs with mutation in the *RB1* gene, which results in a frameshift with premature stop codon. Alterations in genes that are associated with the retinoblastoma pathway is a predictor of poor chance of survival in gliomas [63]. Interestingly, the somatic mutation pattern found in the tumor biopsy from this patient showed mutation of the *IDH1* gene, a common feature of lower grade gliomas [64], which was not detected in the CTC sample. However, CTCs could represent a minority of subclones in the primary tumor, which are not detectable by current standard NGS methods [65].

A *TP53* mutation was found in the CTCs from both patient 3 (GBM) and 4 (anaplastic oligodendroglioma). *TP53* encodes the p53 tumor suppressor protein, and this pathway is often deregulated in diffuse gliomas [66]. Another detected mutation in patient 3 was a missense mutation in the *EPM2AIP1* gene. The *EPM2AIP1* mutations have previously been described in different gastrointestinal cancers [67,68]. Interestingly, *EPM2AIP1* is part of a bidirectional promotor with *MLH1* and epimutations causing hypermethylation has been linked to hereditary colorectal cancers [69,70]. However, little is known regarding the functional role of *EPM2AIP1* silencing, as research has primarily focused on *MLH1*. In patient 4 the WES analysis also detected mutations in the *ALK* gene, which encodes a receptor tyrisone kinase. *ALK* is frequently mutated in neuroblastoma and indeed the detected NM_004304.4_p.R1275L variant is a described hot spot locus within the kinase domain. This hotspot mutation hinders the auto-inhibition of *ALK* and acts transformative. Consequently, neuroblastoma patients with *ALK* mutations show poorer overall survival [71]. Importantly, small molecules for targeted therapy of *ALK* have been developed and neuroblastoma cell lines harboring p.R1275 mutations show sensitivity towards *ALK* inhibitors, such as crizotinib [72,73]. Altogether, the specific detection of glioma-related mutation patterns in the CTC samples strongly indicates that the detected cells originate from a glioma site.

In summary, we present a method for enriching and staining CTCs from glioma patients. After a complete clinical validation the method could provide a powerful tool for non-invasive pheno- and genotyping of gliomas. Finally, the technology could potentially be used to monitor progression and recurrence in cancer patients.

## Figures and Tables

**Table 1:** rVAR2-based protein pull-down hits from cell lysates.

Protein Name	Gene	Peptides Count	Seq. Coverage (%)	Ratio to Neg
**KNS-42**				
Amyloid-like protein 2	APLP2	17	27	NA
CD44	CD44	9	37.4	41.17
Glypican 1	GPC1	10	23.7	NA
Glypican 4	GPC4	10	23	7.15
Integrin beta 1	ITGB1	11	15.2	7.50
Neuropilin 1	NRP1	12	21.1	NA
Neuropilin 2	NRP2	4	6	NA
Receptor-type tyrosine-protein phosphatase zeta	PTPRZ1	17	8.6	NA
Syndecan 1	SDC1	6	14.5	NA
Syndecan 2	SDC2	5	25.5	NA
Testican 1	SPOCK1	8	22.6	NA
Versican	VCAN	39	12.8	540.37
**U118mg**				
Amyloid-like protein 2	APLP2	4	5.2	NA
CD44	CD44	8	37.4	17.26
Decorin	DCN	8	29.8	41.89
Neuropilin 1	NRP1	9	15.9	16.74
Versican	VCAN	13	4	NA
**U87mg**				
Amyloid-like protein 2	APLP2	4	5.1	NA
Amyloid precursor protein	APP	3	4.3	NA
Carbonic anhydrase 9	CA9	1	3.3	NA
CD44	CD44	4	6.1	12.92
Chondroitin sulfate proteoglycan 4	CSPG4	7	3.1	NA
HLA class II histocompatibility antigen gamma chain	CD74	3	12.2	NA
Sushi repeat-containing protein SRPX	SRPX	7	15.7	NA
Syndecan-1	SDC1	3	15.2	9.71
Syndecan-4	SDC4	2	12.6	NA

**Table 2:** Mutations detected in patient CTCs by whole exome sequencing (WES).

Patient Information						Confirmed Mutation by WES					
ID	Diagnosis	Molecular Features in Tumor Biopsy	Sample	CTCs	WBCs	Gene	Transcript ID	Transcript Variant	Allele Fraction (%)	Protein Variant	Translation Impact
**1**	Anaplastic oligodendro-glioma	IDH1 mutation LOH 1p/19q MGMT methylation	1	2	19	RB1	NM_000321.2	c.1644delA	16.88	p.K548fs*3	Frameshift
			2	3	34	RB1	NM_000321.2	c.1644delA	1.59	p.K548fs*3	Frameshift
**3**	GBM	IDH1 wild type	1	2	23	TP53	NM_000546.5	c.892G>T	21.39	p.E298*	Stop-gain
						EPM2AIP1	NM_014805.3	c.128G>T	54.17	p.R43L	Missense
**4**	Anaplastic oligodendro-glioma	IDH1 mutation 1p/19q deletion MGMT methylation	1	4	14	TP53	NM_000546.5	c.493C>T	31.43	p.Q165*	Stop-gain
						ALK	NM_004304.4	c.3824G>T	33.33	p.R1275L	Missense

## Supplementary Materials

The following are available online at https://www.mdpi.com/2073-4409/8/9/998/s1, Figure S1: Proximity ligation assay controls, Figure S2: ofCS expression on glioma cell lines, Figure S3: rVAR2 staining of rVAR2-captured glioma cells, Figure S4: Screenshots from the Integrative Genomic Viewer, Figure S5: Images of detected patient CTCs, Figure S6: In vitro EMT western blot.
