# Supplementary material for: Capture and Detection of Circulating Glioma Cells Using the Recombinant VAR2CSA Malaria Protein
Source: Cells. 2019 Aug 28;8(9):998. doi: 10.3390/cells8090998 (PMC6769911; doi:10.3390/cells8090998)

Supplementary figure S1. Proximity ligation assay controls

Quantification of the proximity ligation assay (PLA) co-localization signals between rVAR2 or DBL4 (negative control) and each of the CSPGs analyzed. Data is shown as the number of signals per cell. Red bars represent the mean number of signals per cell. The figure shows the results obtained for U87mg (A), U118mg (B), and KNS-42 (C).

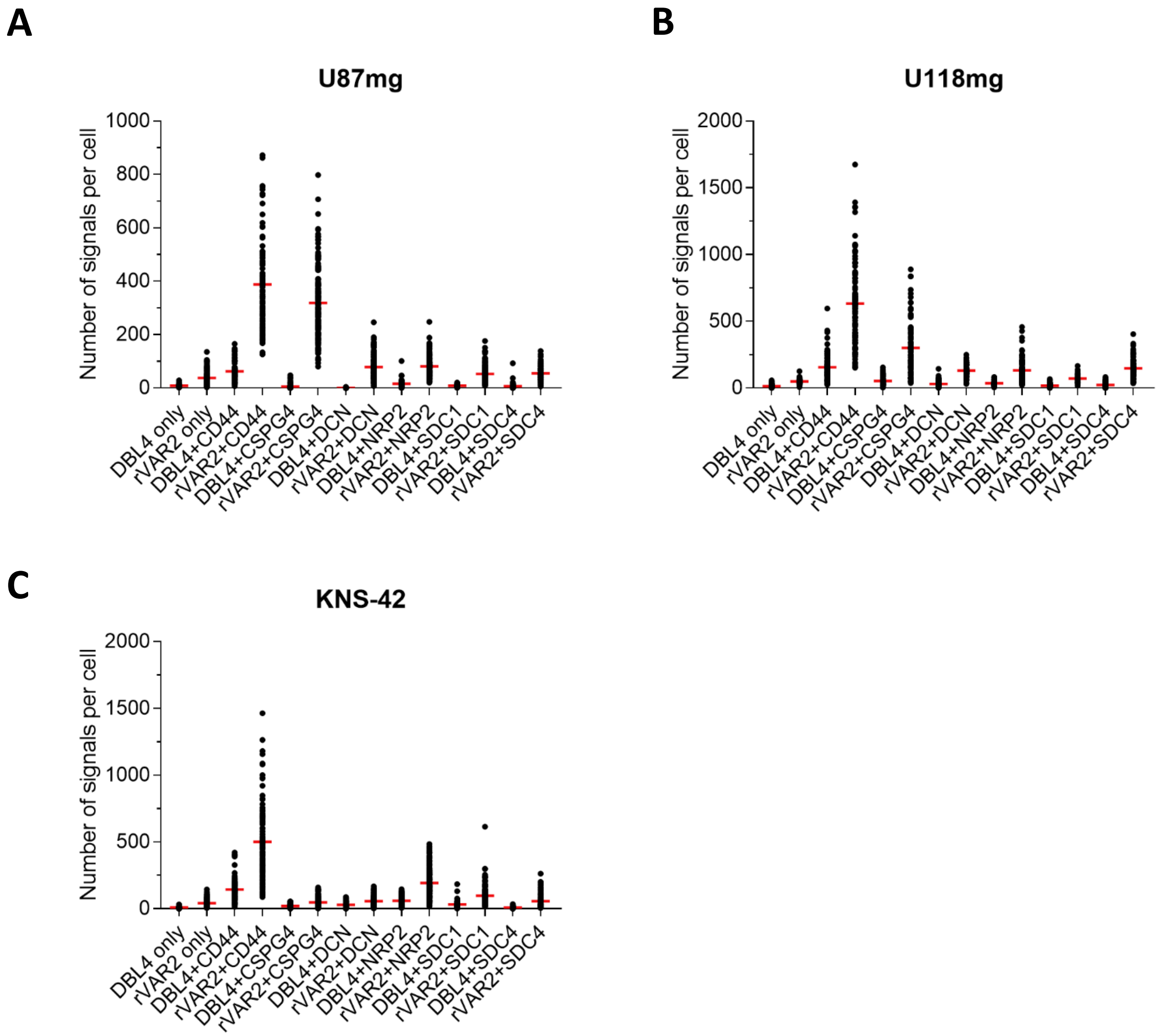

Supplementary figure S2. ofCS expression on glioma cell lines

rVAR2 binding to the glioma cell lines KNS-42, Res259, U87mg and U118mg (A) or Res259, U87mg and U118mg (B) was measured by flow cytometry using a FITC-conjugated anti-V5 antibody. Geometric Mean Fluorescence Intensity (MFI) was measured after incubation of cells with various rVAR2 concentrations. Results are displayed as signal/noise ratio. Each panel represents an independent experiment.

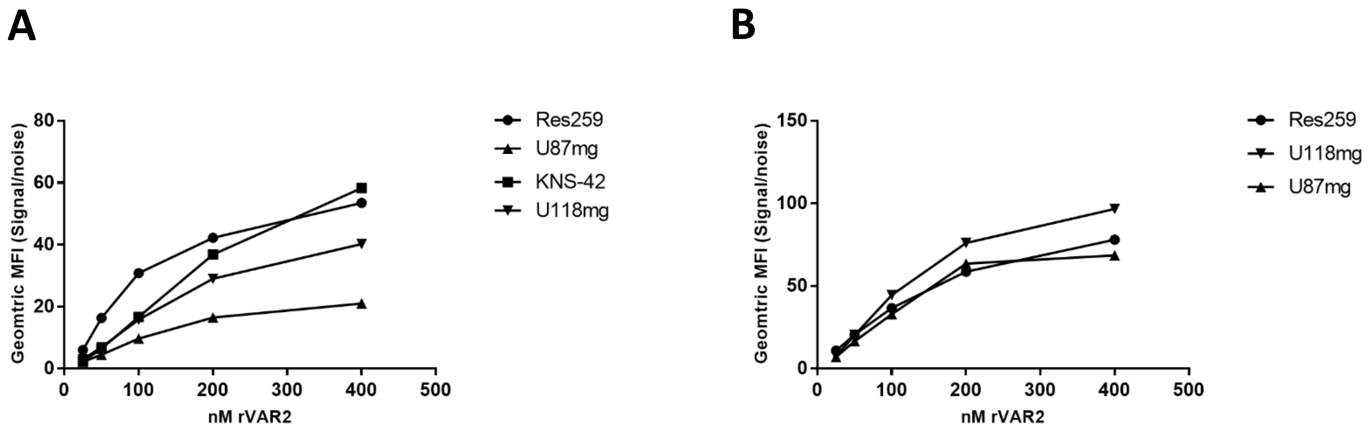

Supplementary figure S3. rVAR2 staining of rVAR2-captured glioma cells

U87mg cells were incubated with rVAR2-coupled beads and stained with a fluorophore-conjugated rVAR2 (green) and DAPI (blue) (A) or control buffer and DAPI (B). Representative images were obtained using the Cytation 3 Imager with a 10x objective. Scale bars, 20μm.

A B

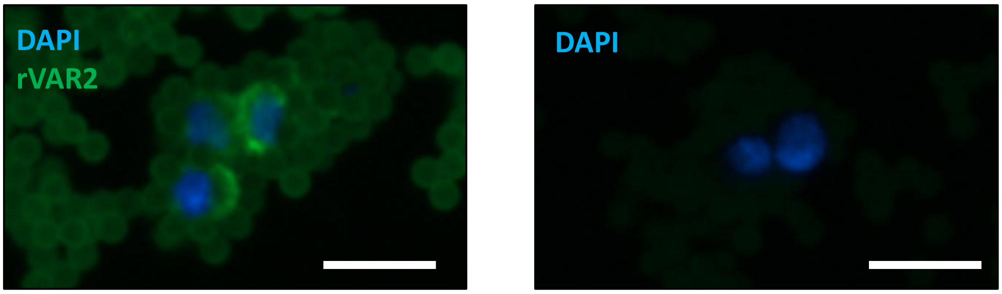

Supplementary figure S4. Screenshots from the Integrative Genomics Viewer

Screen shots from the Integrative Genomics Viewer (Broad Institute, UK) showing aligned read sequences in regions with confirmed cancer-related mutations. Data is based on patient-specific white blood cells as a matched reference sample. The whole exome sequencing data was aligned against the human reference genome (hg19/GRCh37). For patient 1 reads in the region containing the RB1 gene from both samples containing patient CTCs as well as the WBC control are shown (A). For patient 3 reads from the CTC containing sample as well as the WBC control is shown for the TP53 gene (B) and the EPM2AIP1 gene (C). For patient 4 reads from the CTC containing sample as well as the WBC control is shown for the TP53 gene (D) and the ALK gene (E).

A

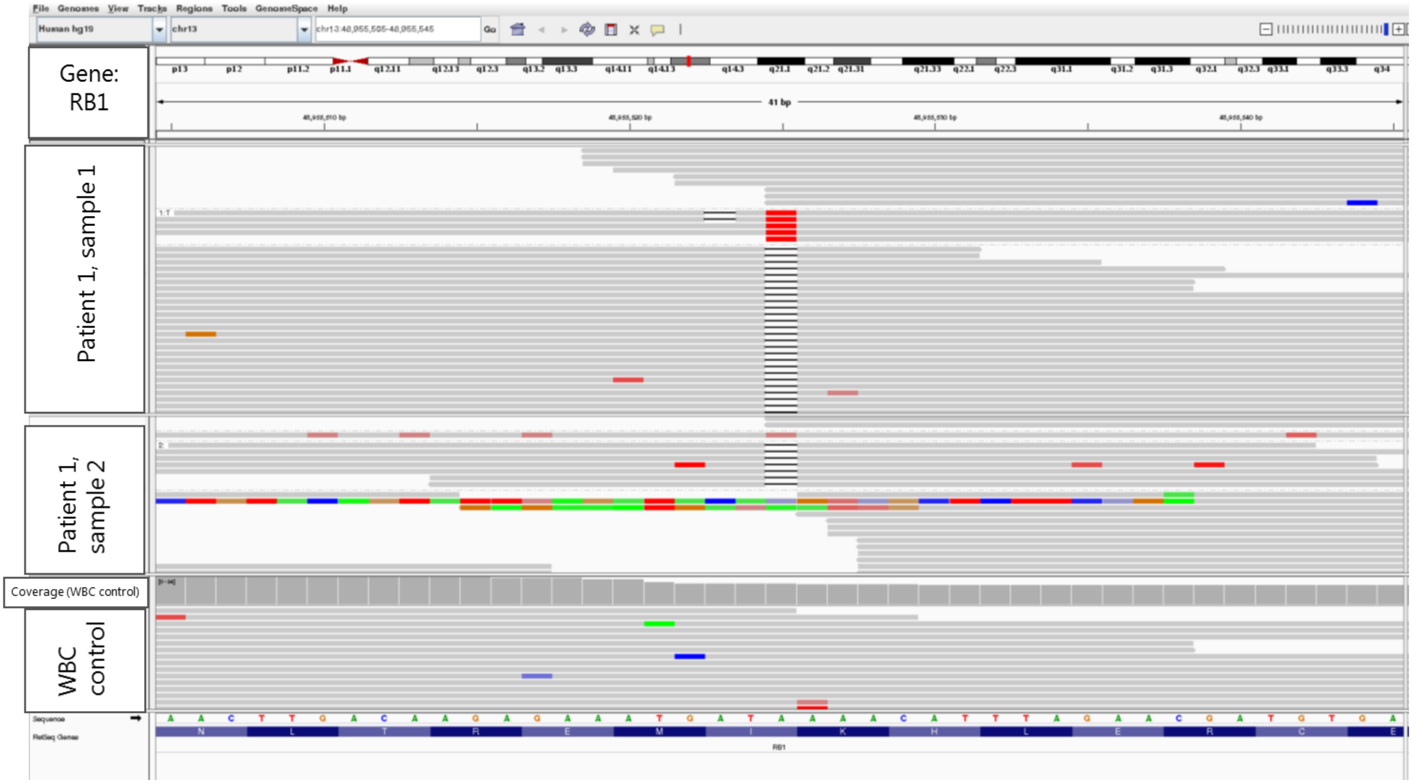

B

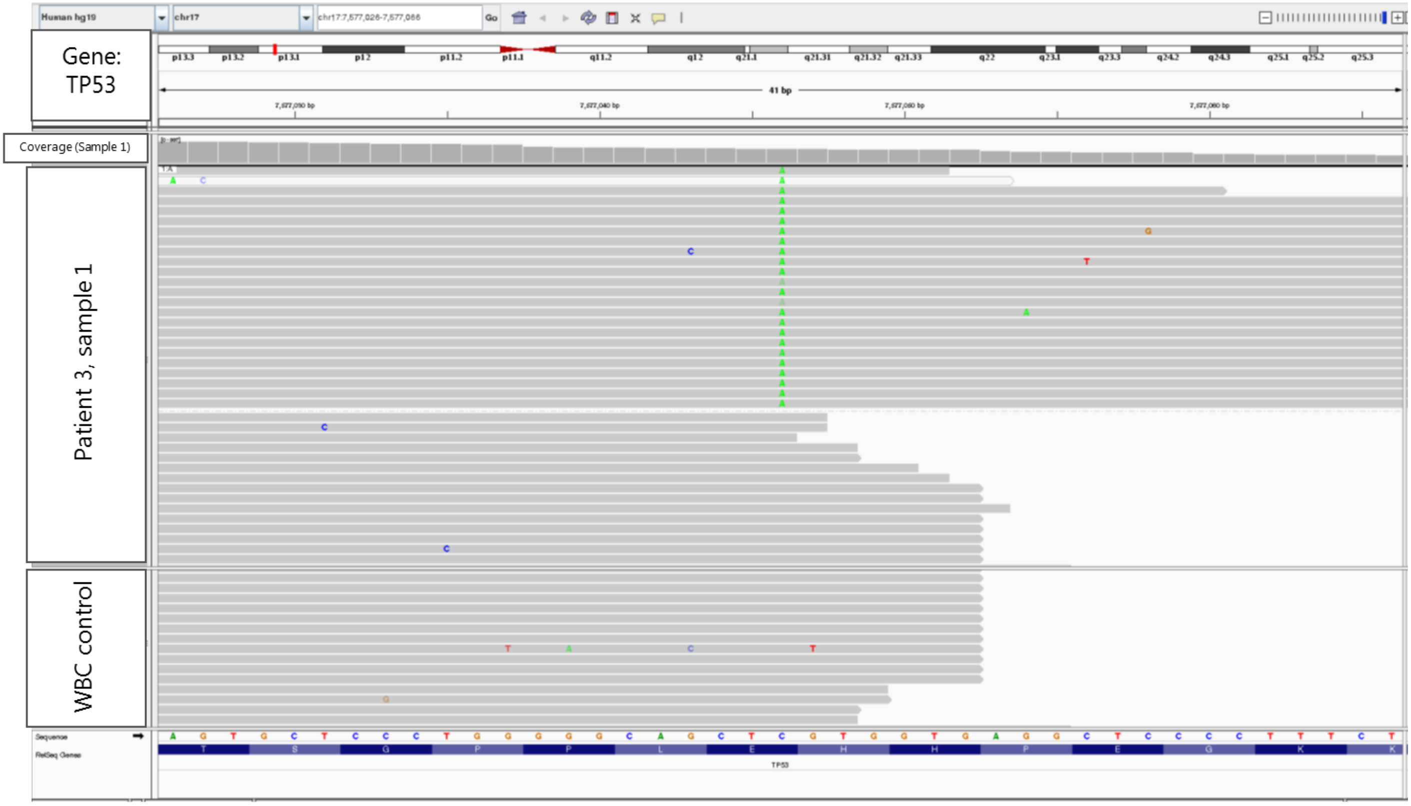
Human hg19
chr17
Gene: TP53
Coverage (Sample 1)
Patient 3, sample 1
WBC control

C

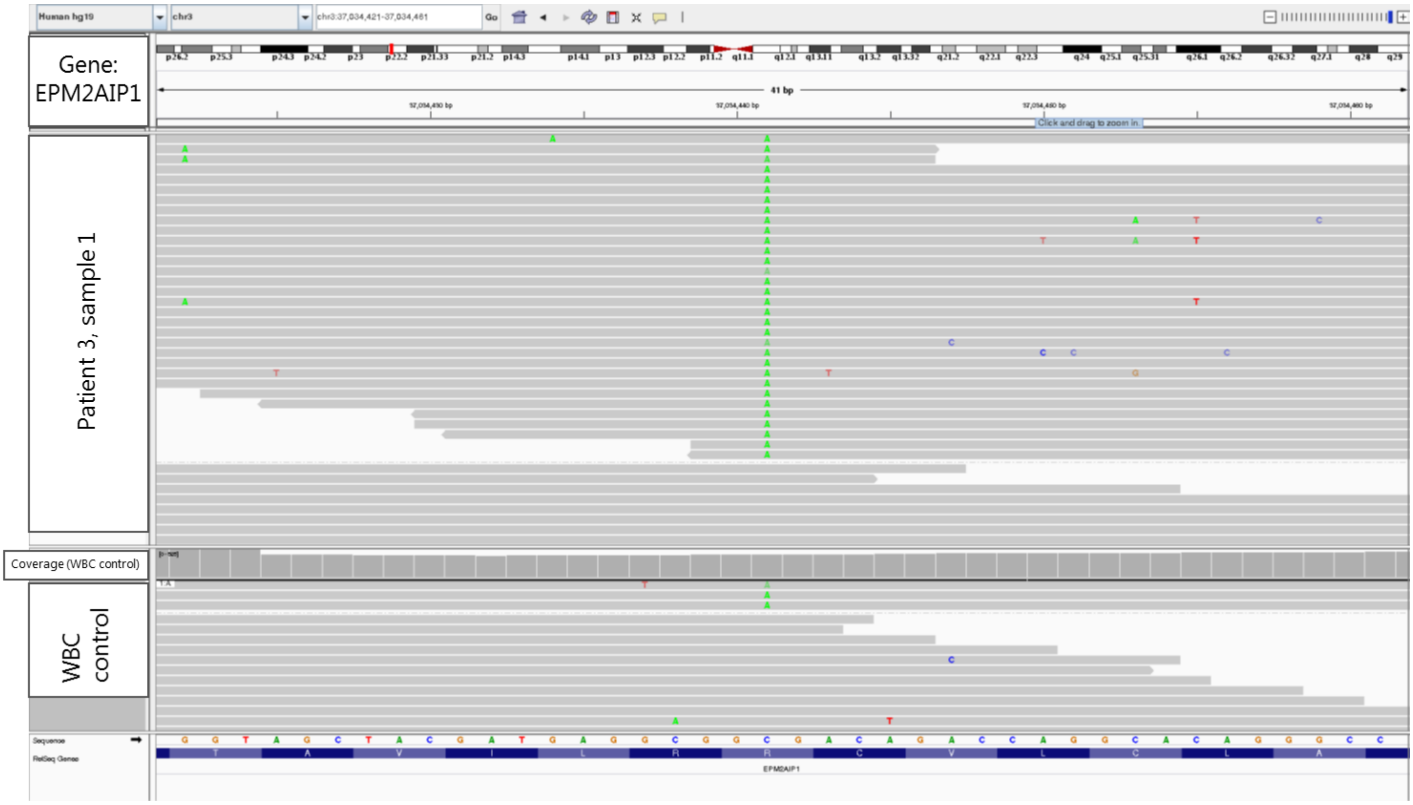
Human hg19
chr3
Gene: EPM2AIP1
Patient 3, sample 1
Coverage (WBC control)
WBC control

D

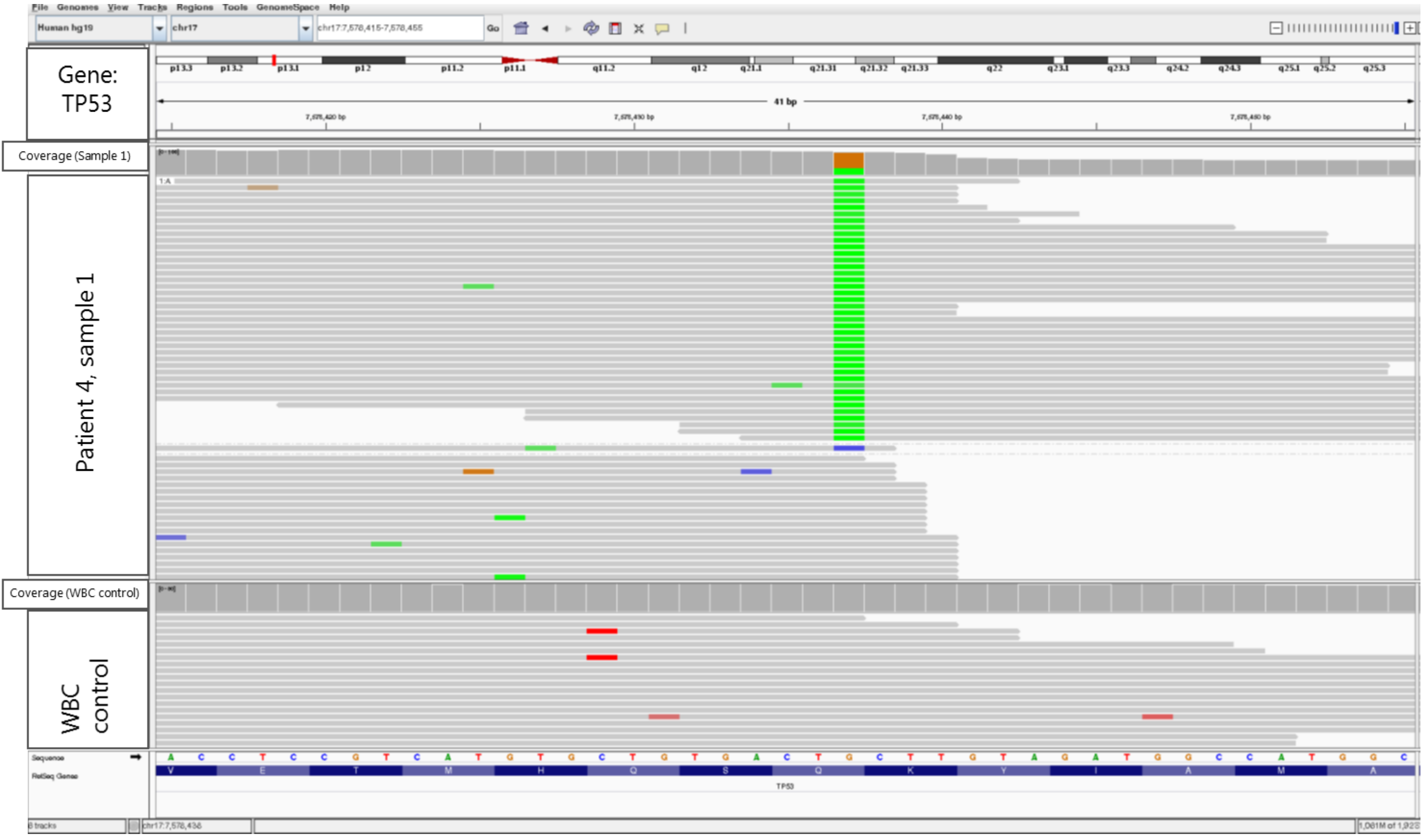
Gene: TP53
Coverage (Sample 1)
Patient 4, sample 1
Coverage (WBC control)
WBC control
TP53

E

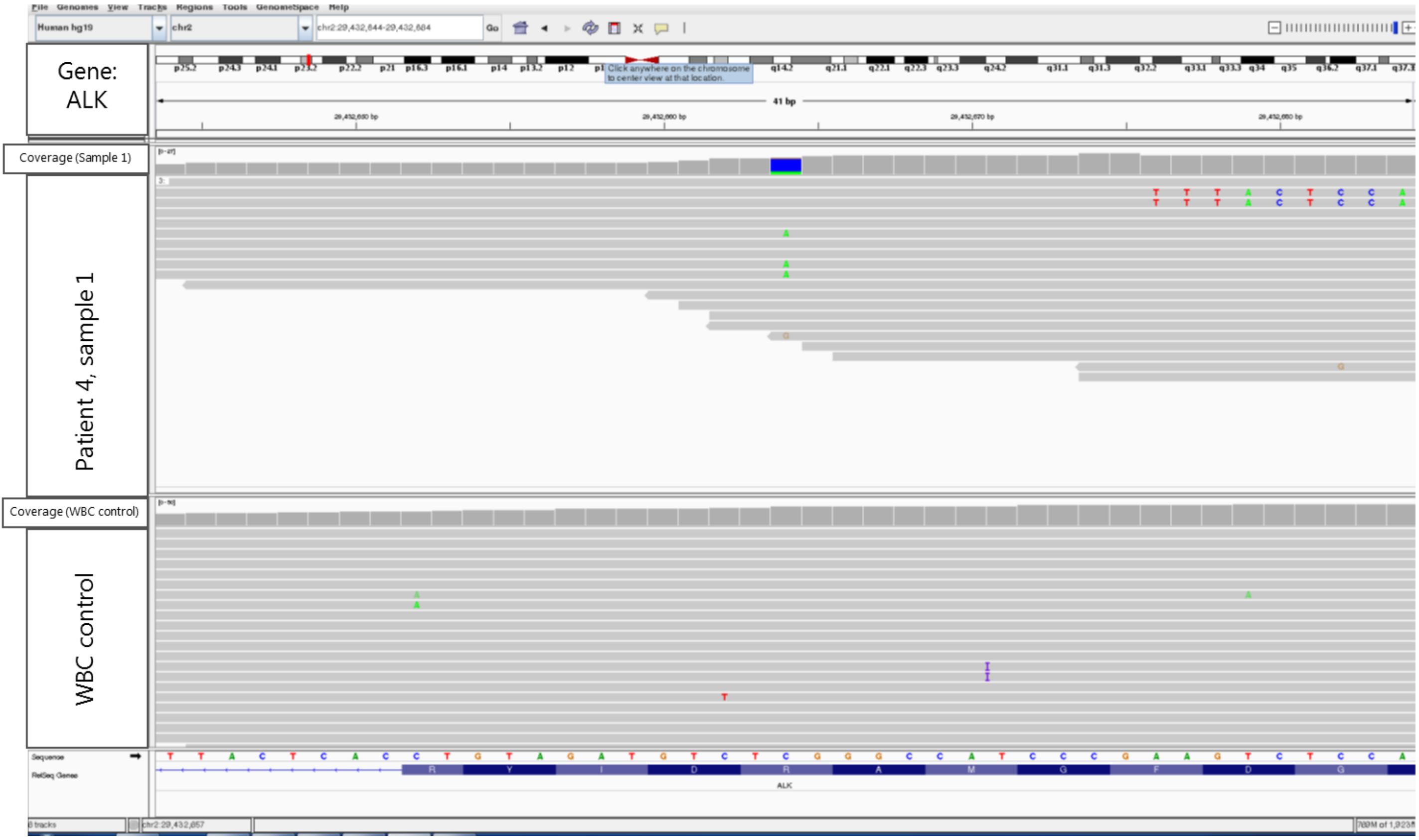
Gene: ALK
Coverage (Sample 1)
Patient 4, sample 1
Coverage (WBC control)
WBC control
ALK

Supplementary figure S5. Images of detected patient CTCs

Images showing detected rVAR2+ positive CD45/CD66b negative cells in all patient samples by manual enumeration using the Gen5 software (BioTek). Images were acquired using the 10X objective on the Cytation™ 3 Cell Imaging Multi-Mode Reader. For all patients 2 x 3 mL blood was processed (here called sample A and B), except for patient 3 where only 3 mL blood was processed. Scale bars, 30 µm.

Patient 1, sample A (3mL)

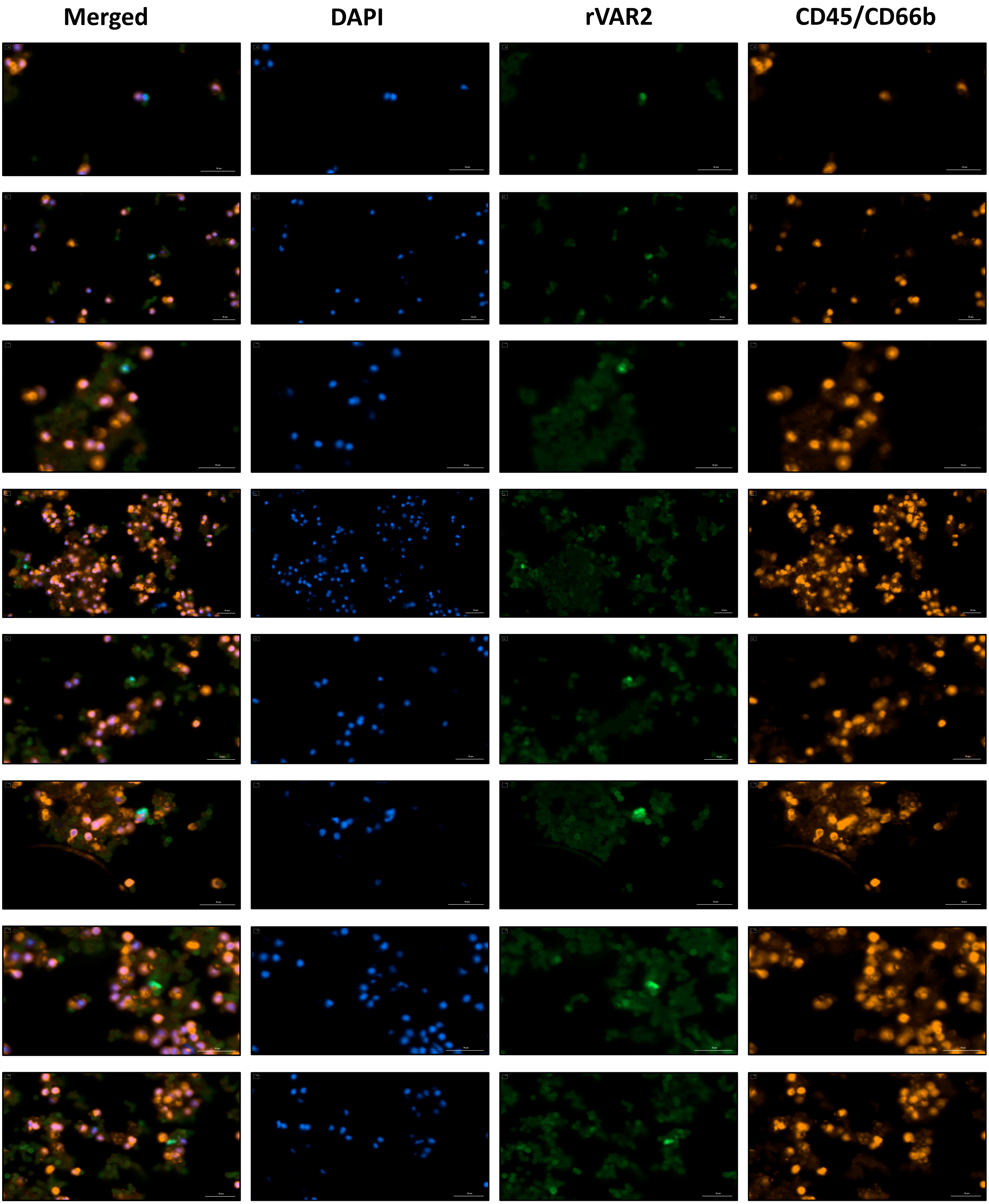

Patient 1, sample A (3mL)

Merged	DAPI	rVAR2	CD45/CD66b

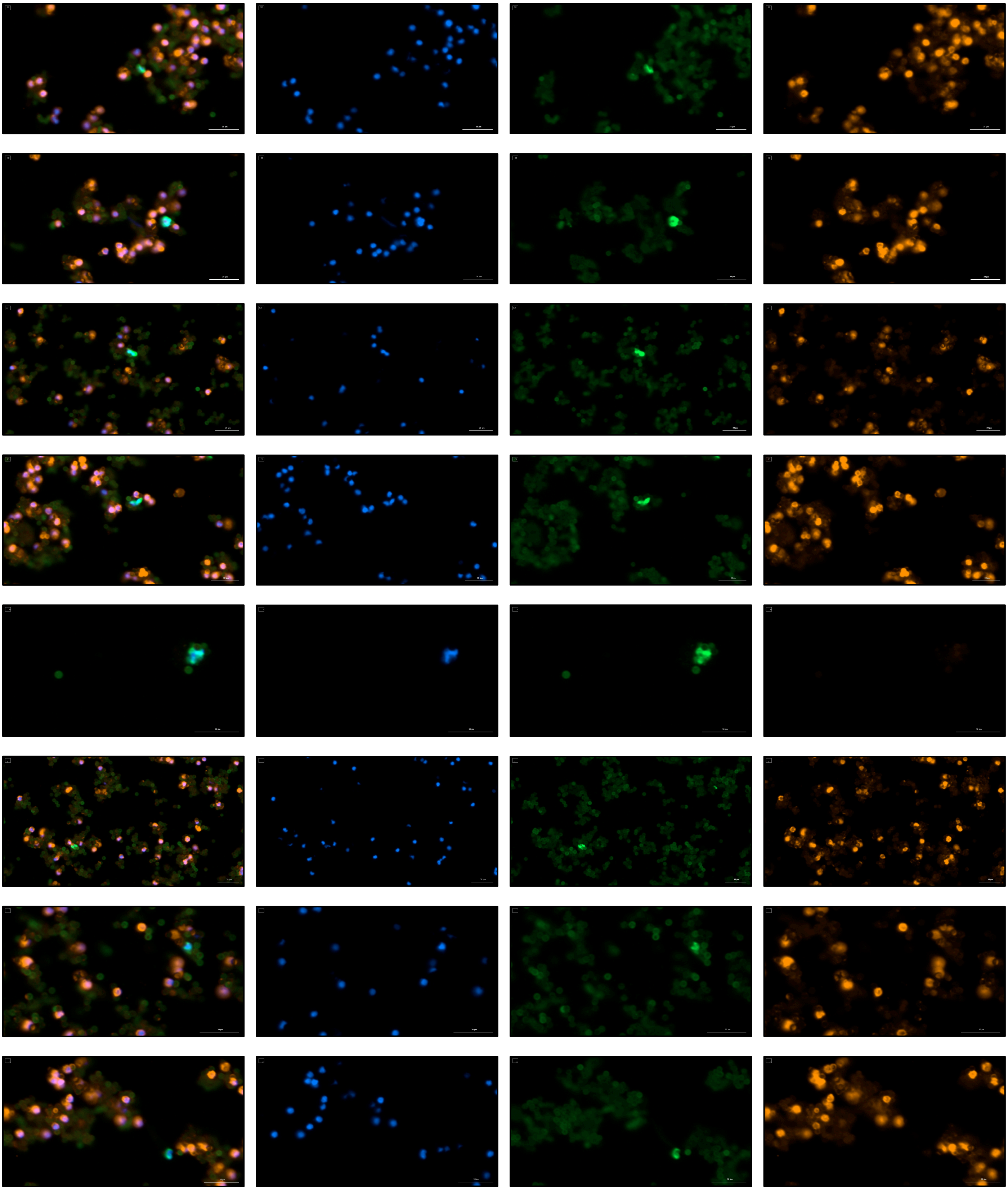

Patient 1, sample A (3mL)

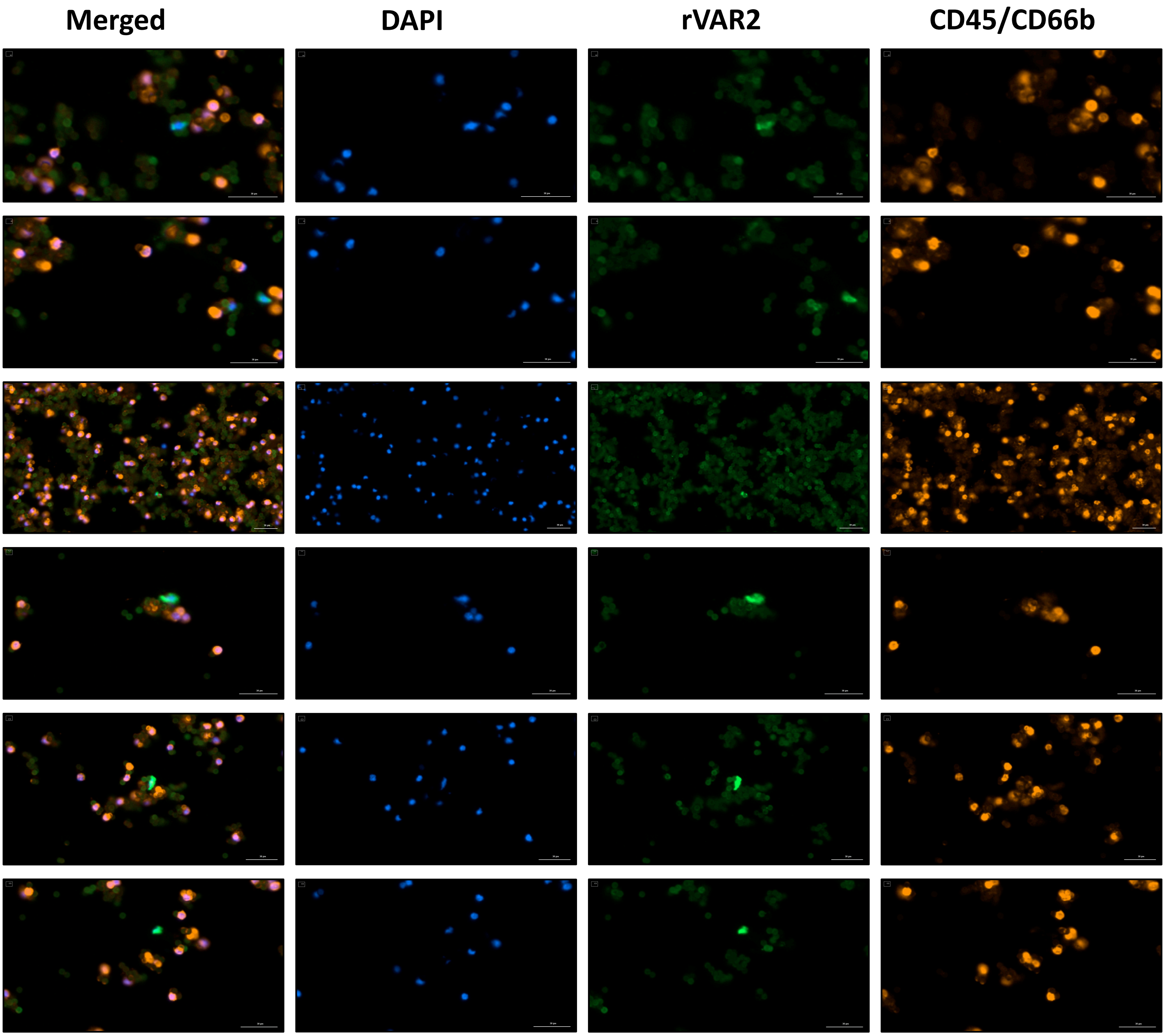

Patient 1, sample B (3mL)

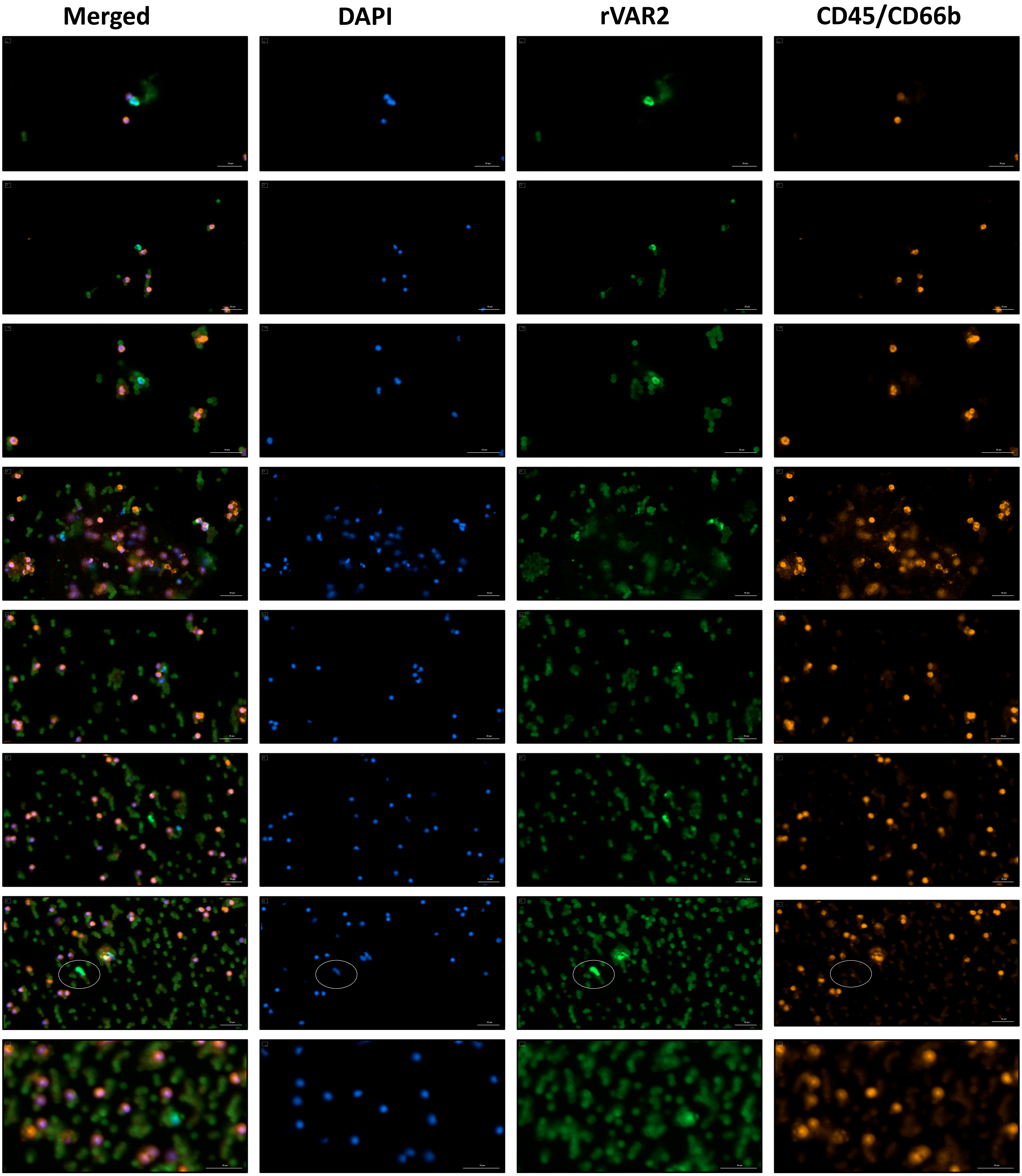

Patient 1, sample B (3mL)

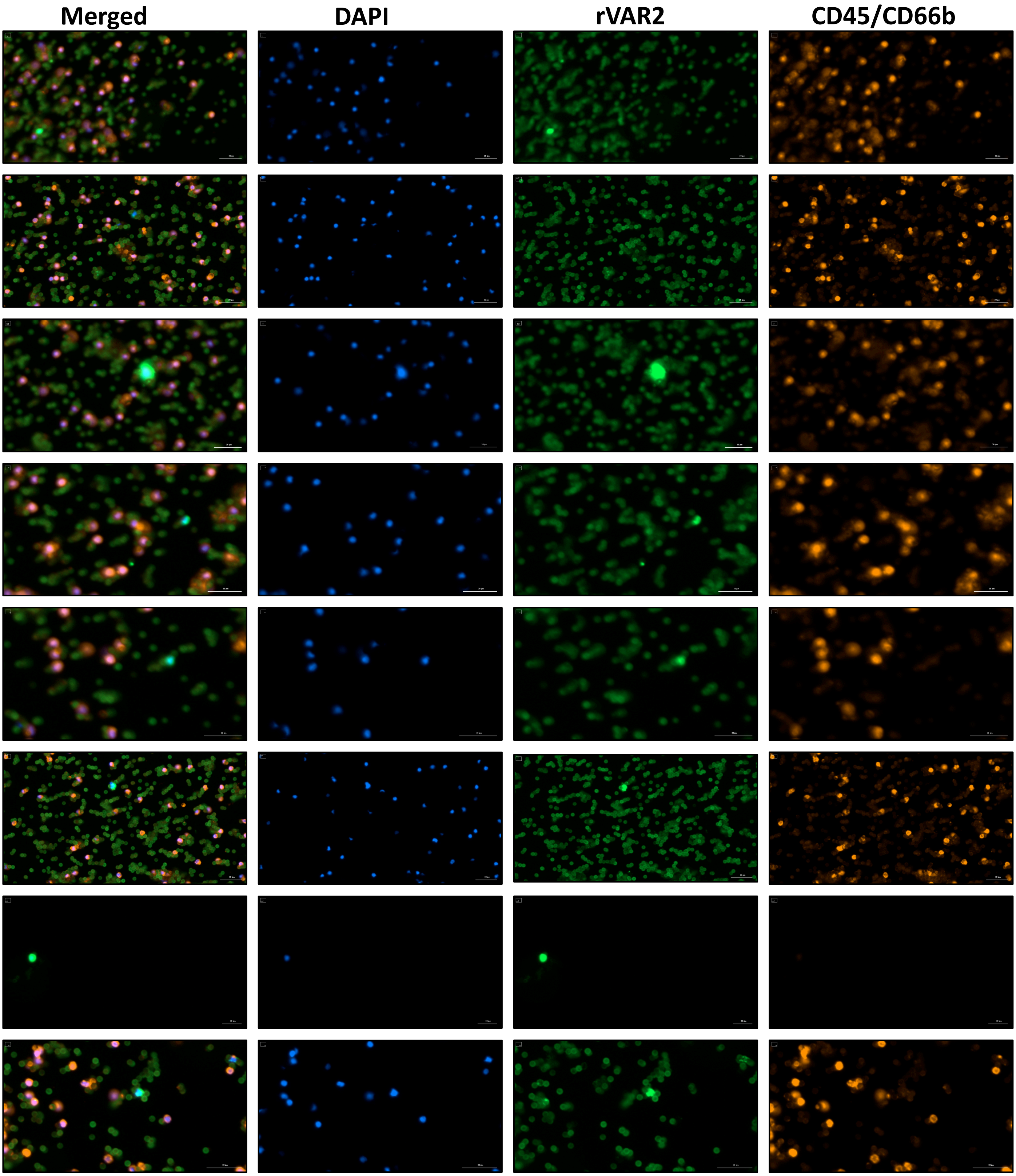

Patient 1, sample B (3mL)

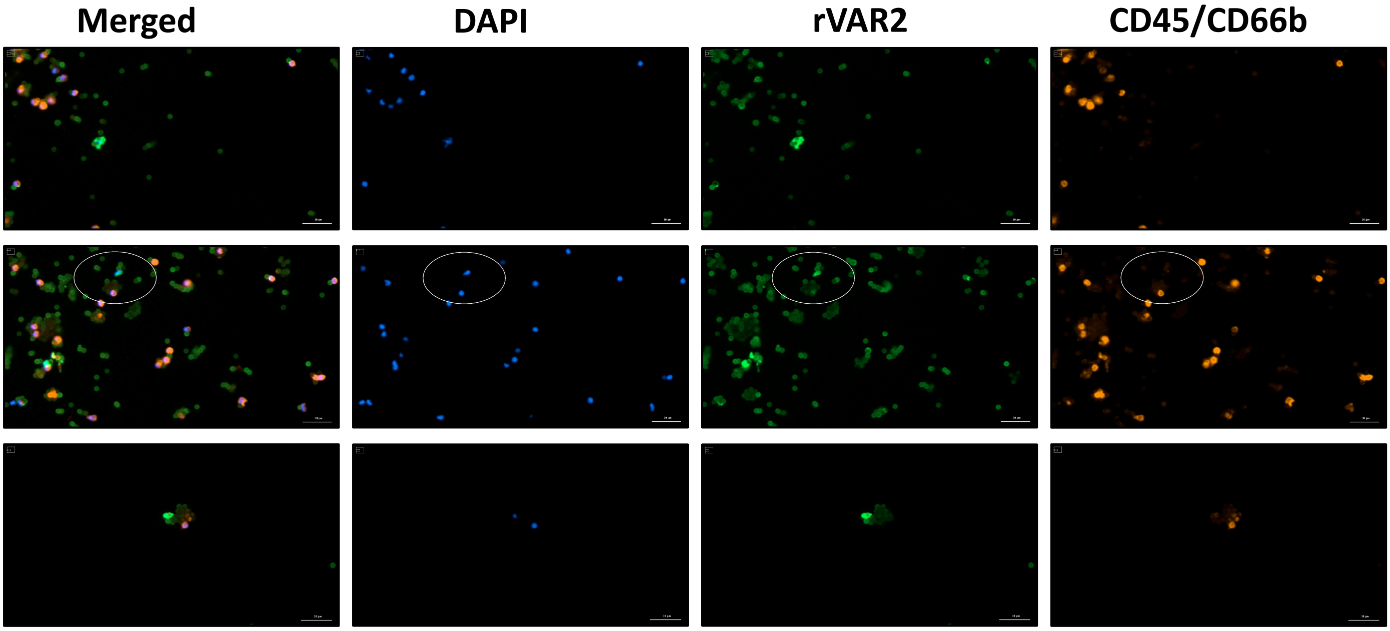

Patient 2, sample A (3mL)

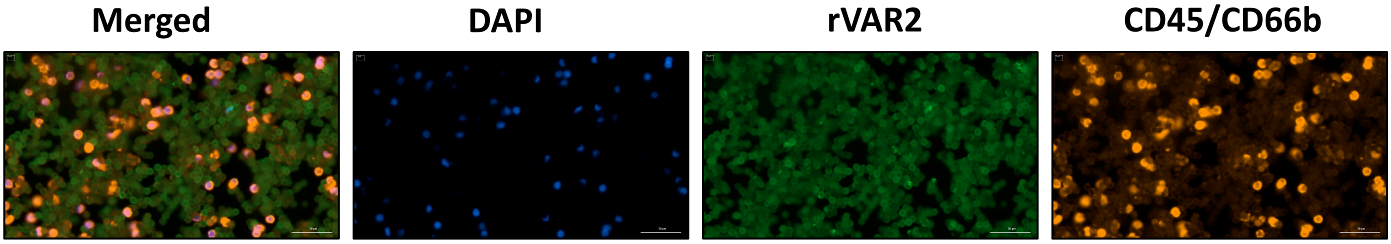

Patient 3, sample A (3mL)

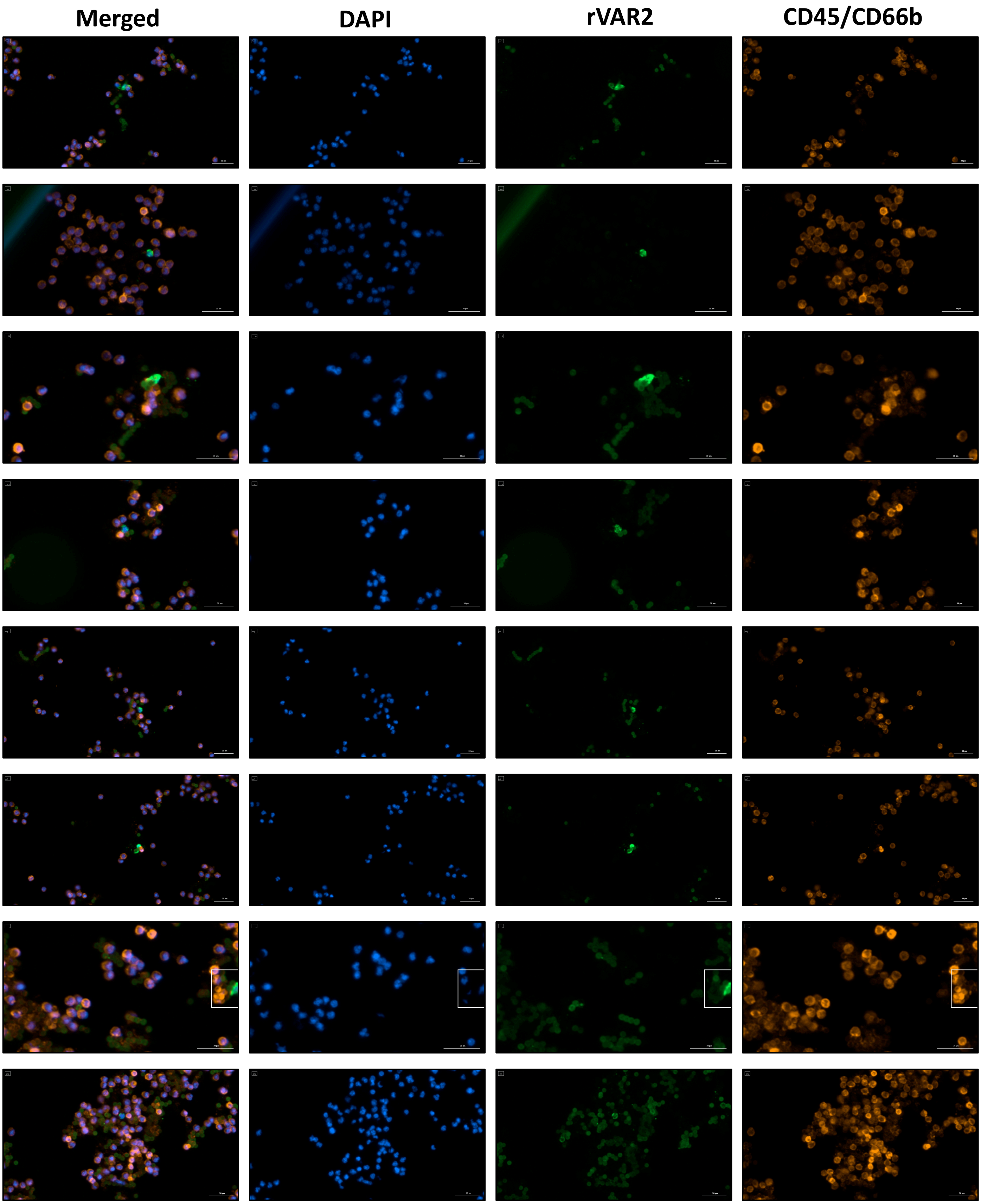

Patient 3, sample A (3mL)

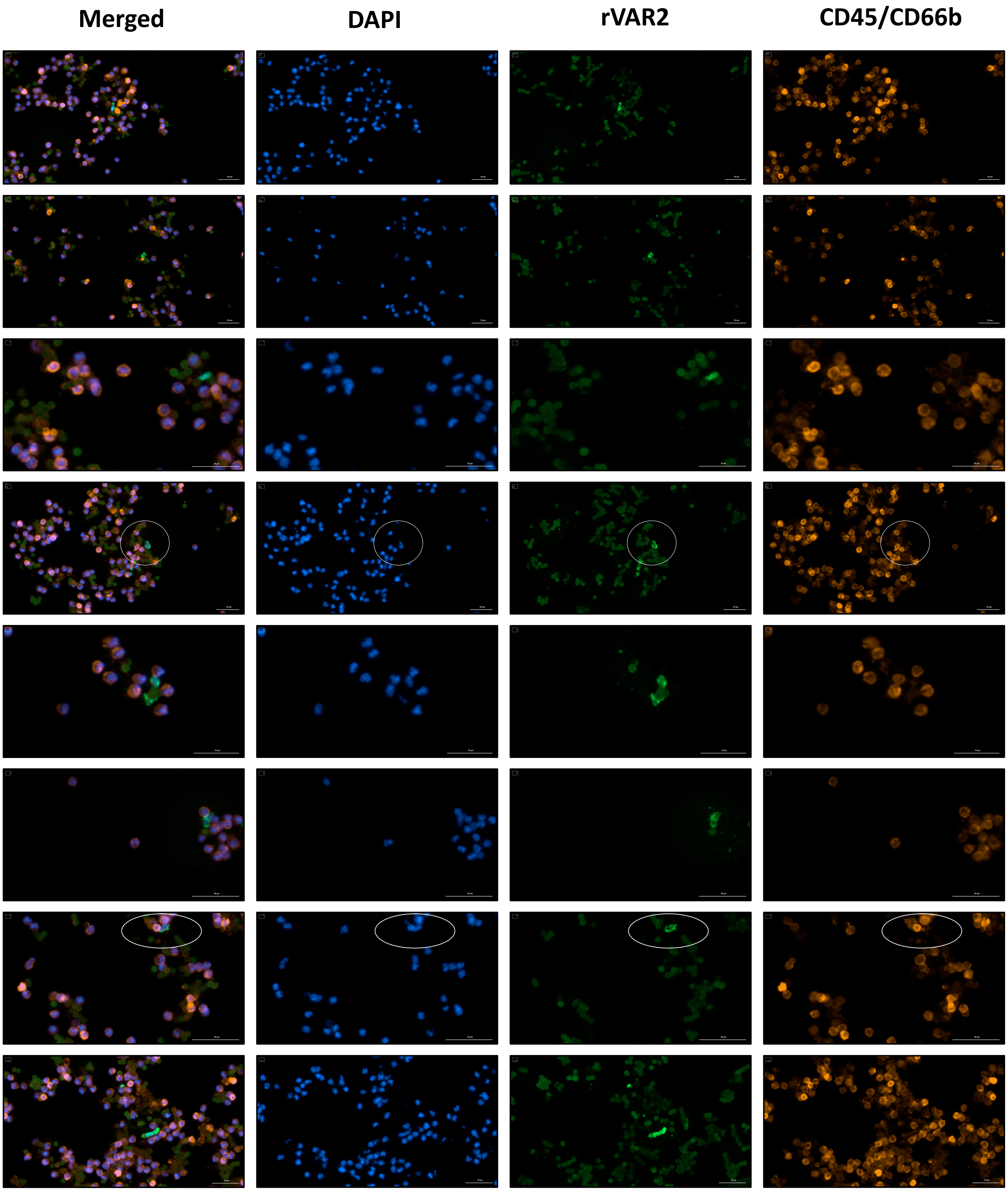

Patient 3, sample A (3mL)

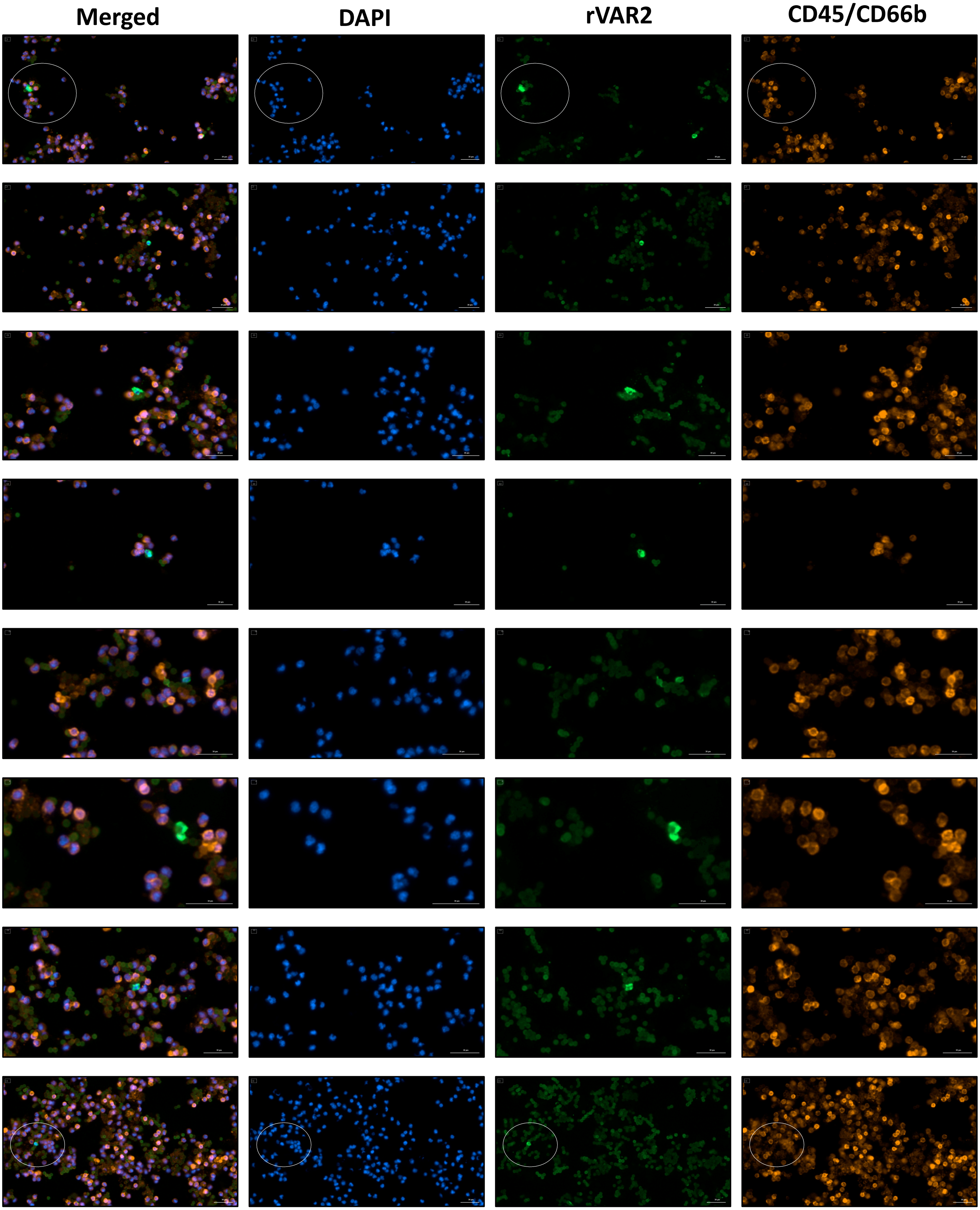

Patient 3, sample A (3mL)

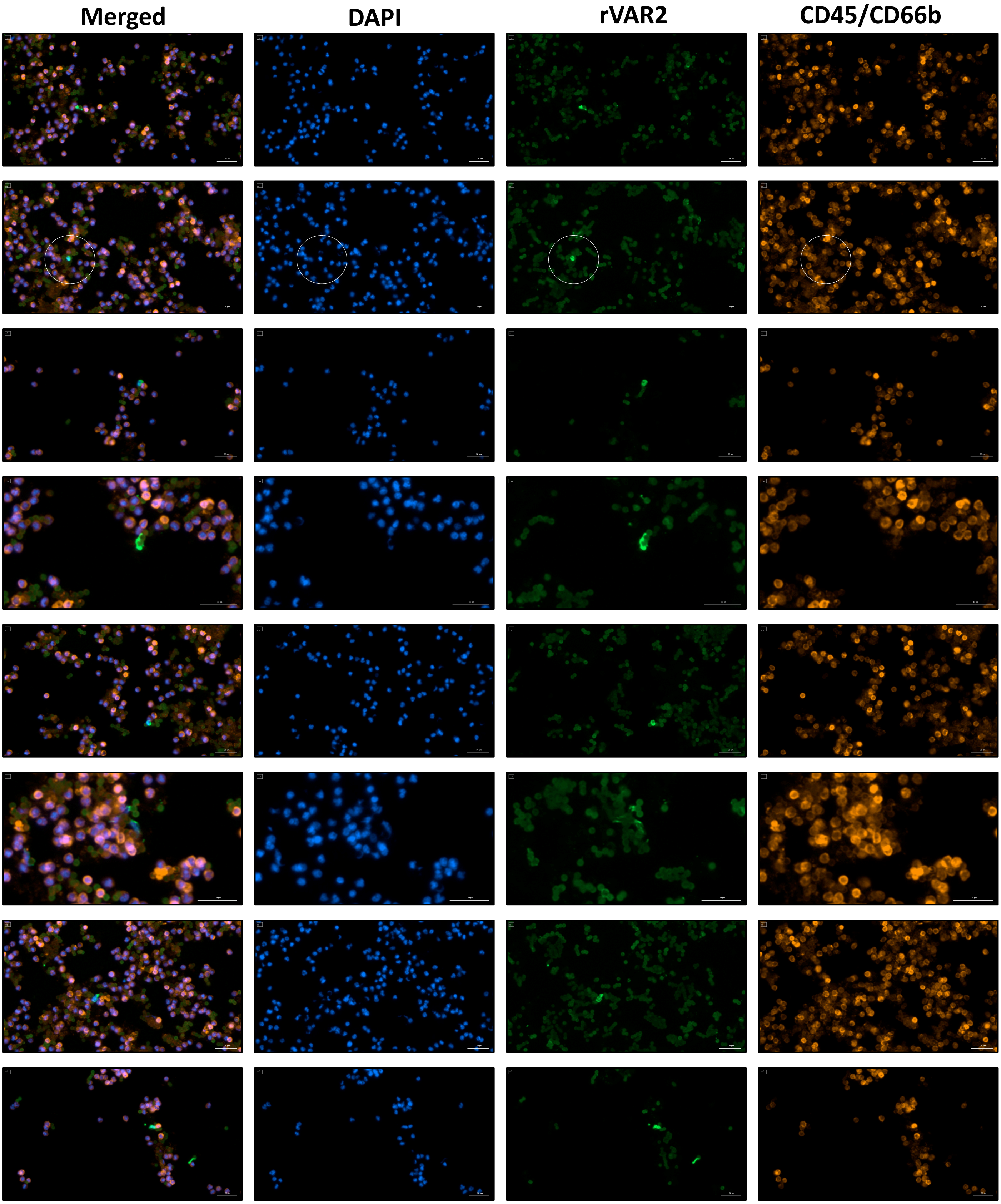

Patient 3, sample A (3mL)

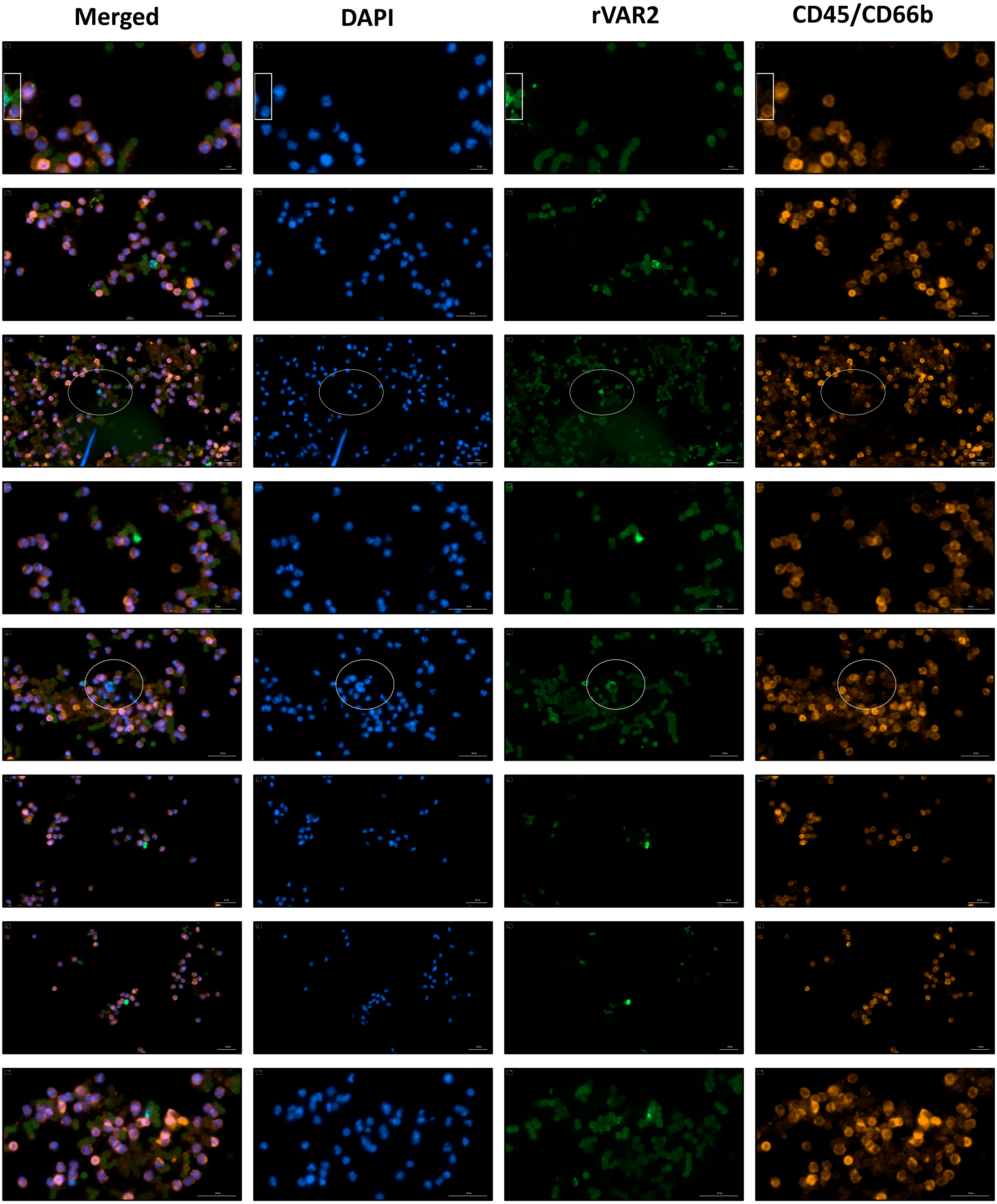

Patient 3, sample A (3mL)

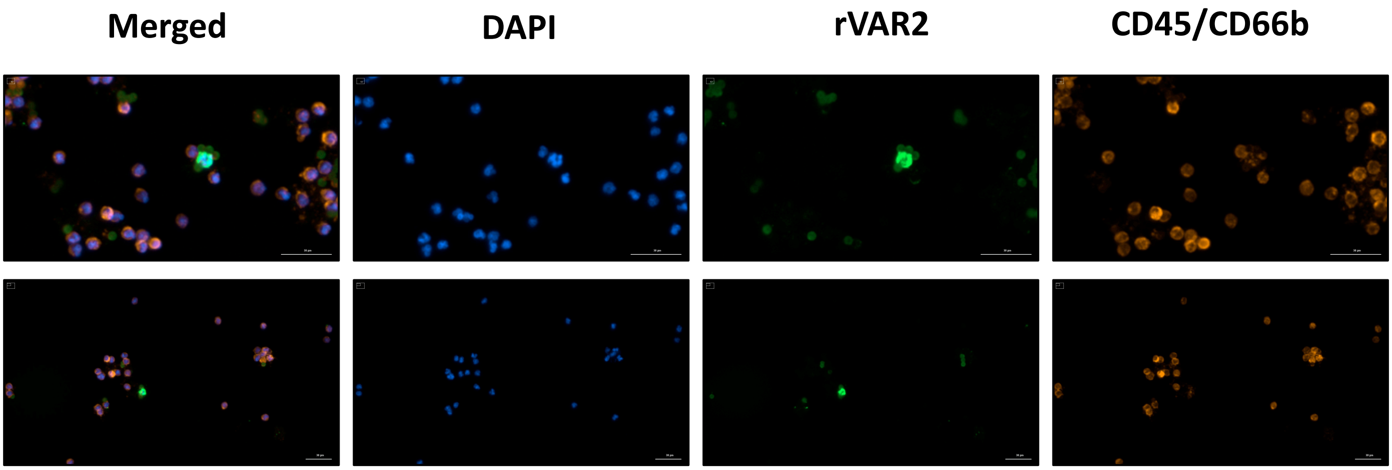

Patient 4, sample A (3mL)

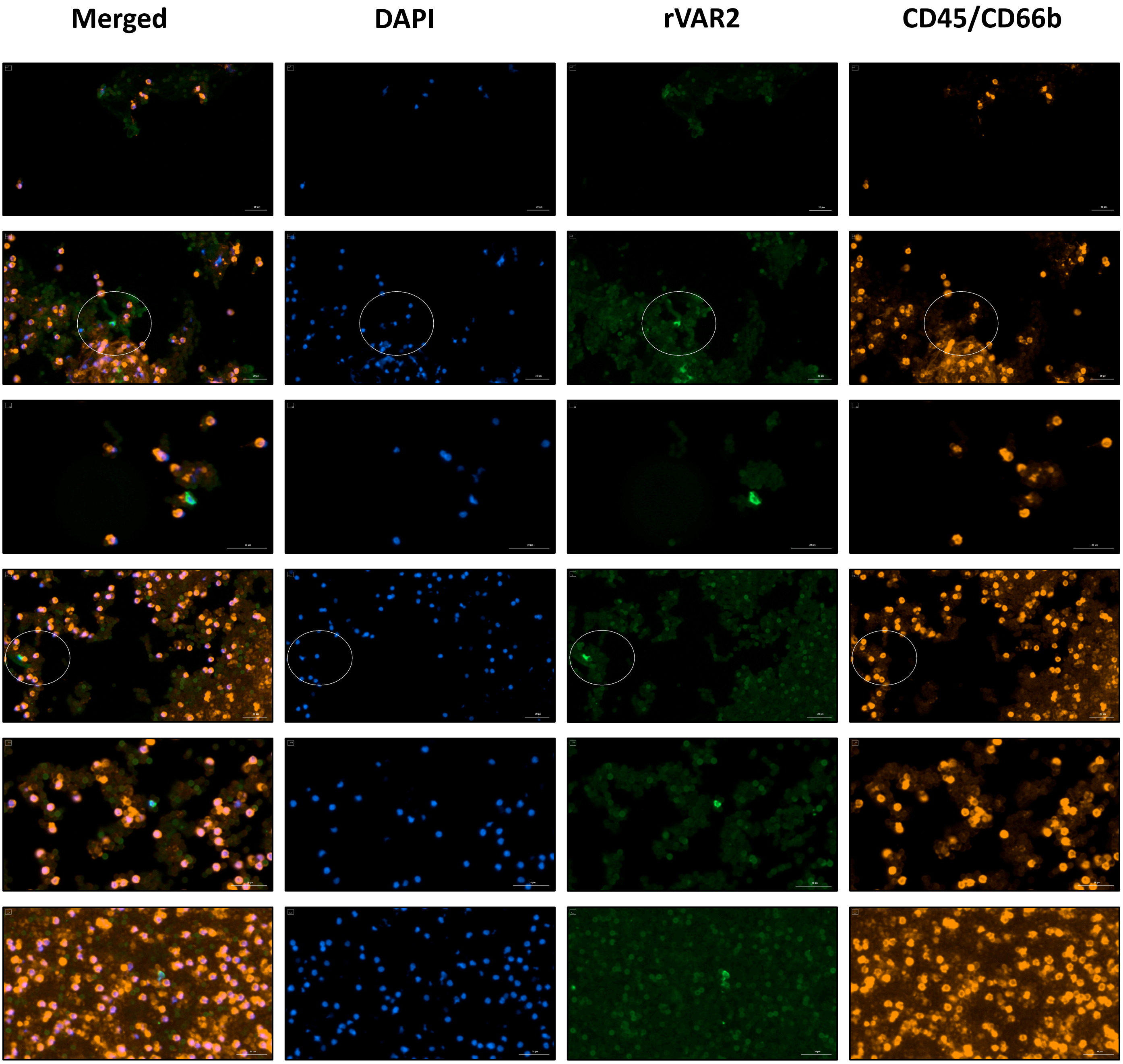

Patient 4, sample B (3mL)

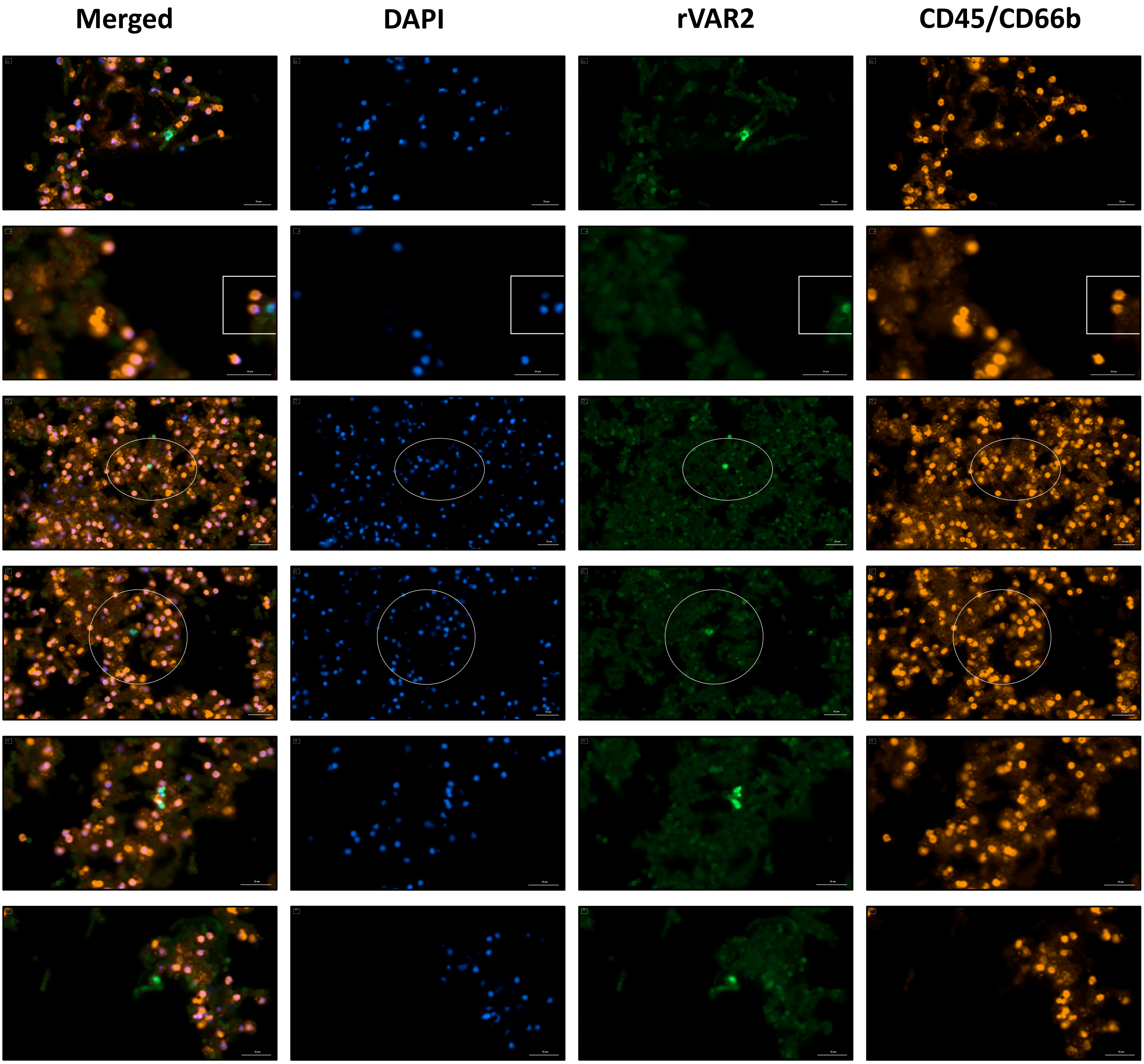

Patient 5, sample A (3mL)

Merged **DAPI** **rVAR2** **CD45/CD66b**

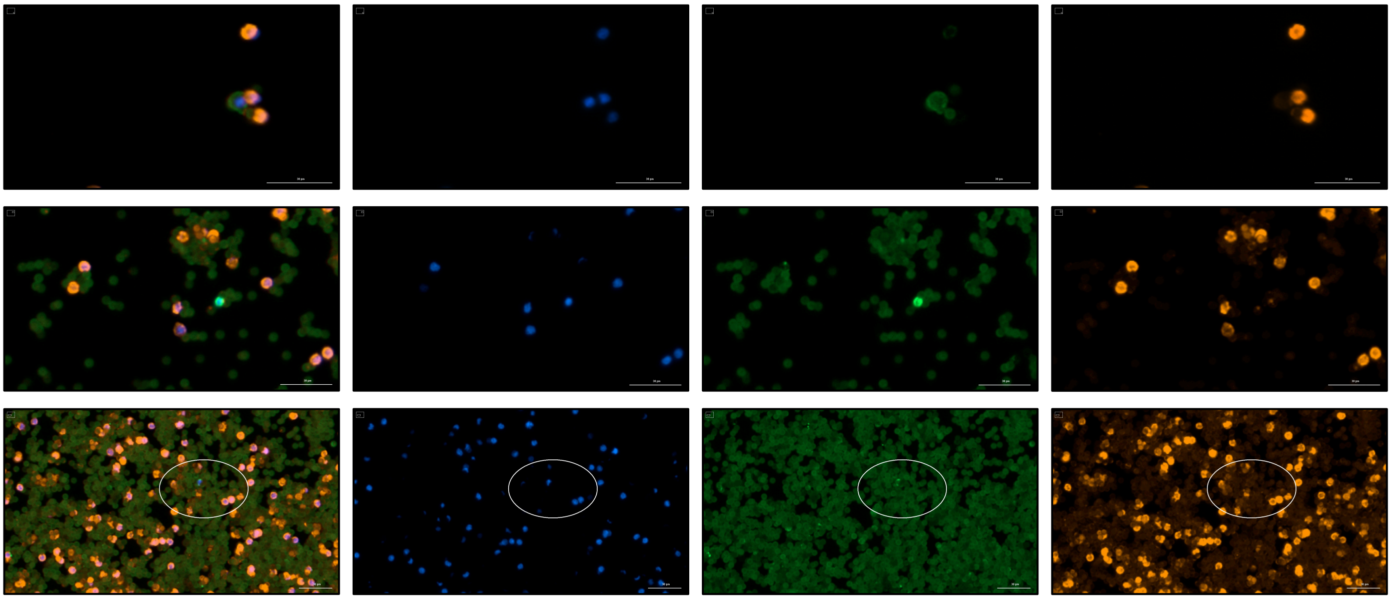

Patient 5, sample B (3mL)

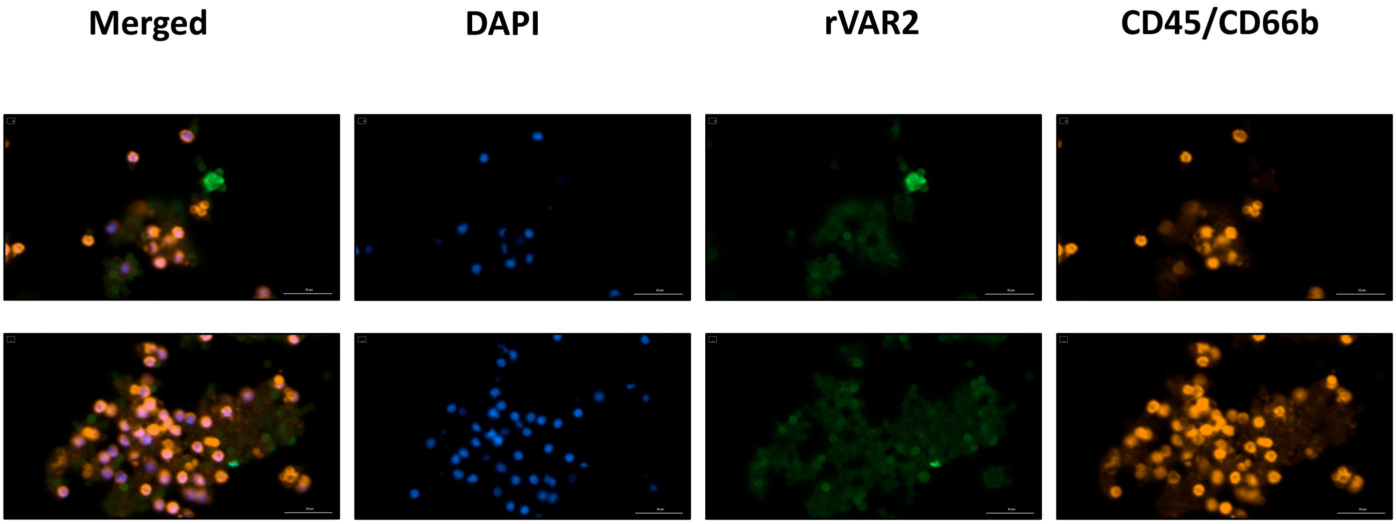

Patient 6, sample B (3mL)

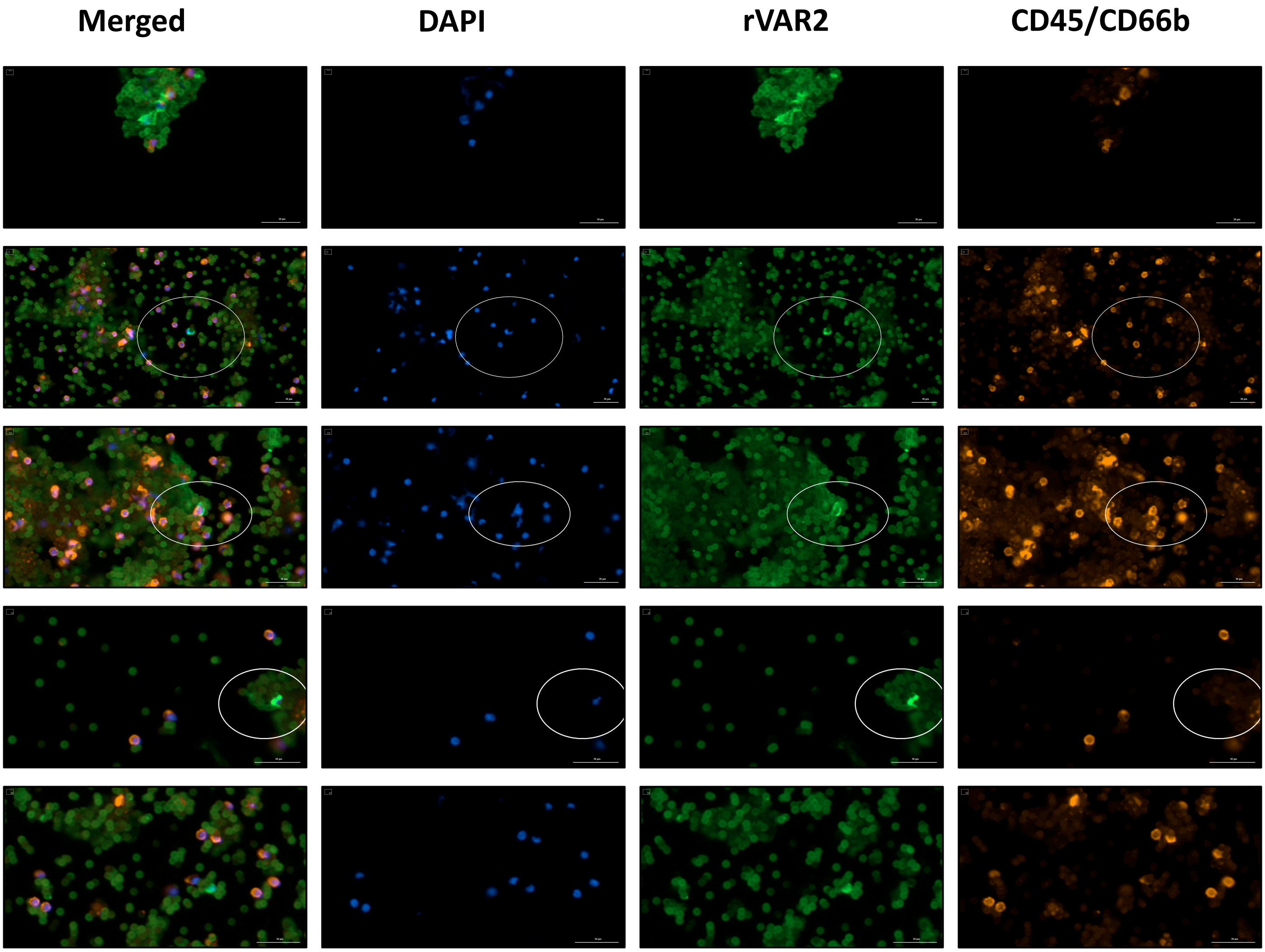

Patient 7, sample A (3mL)

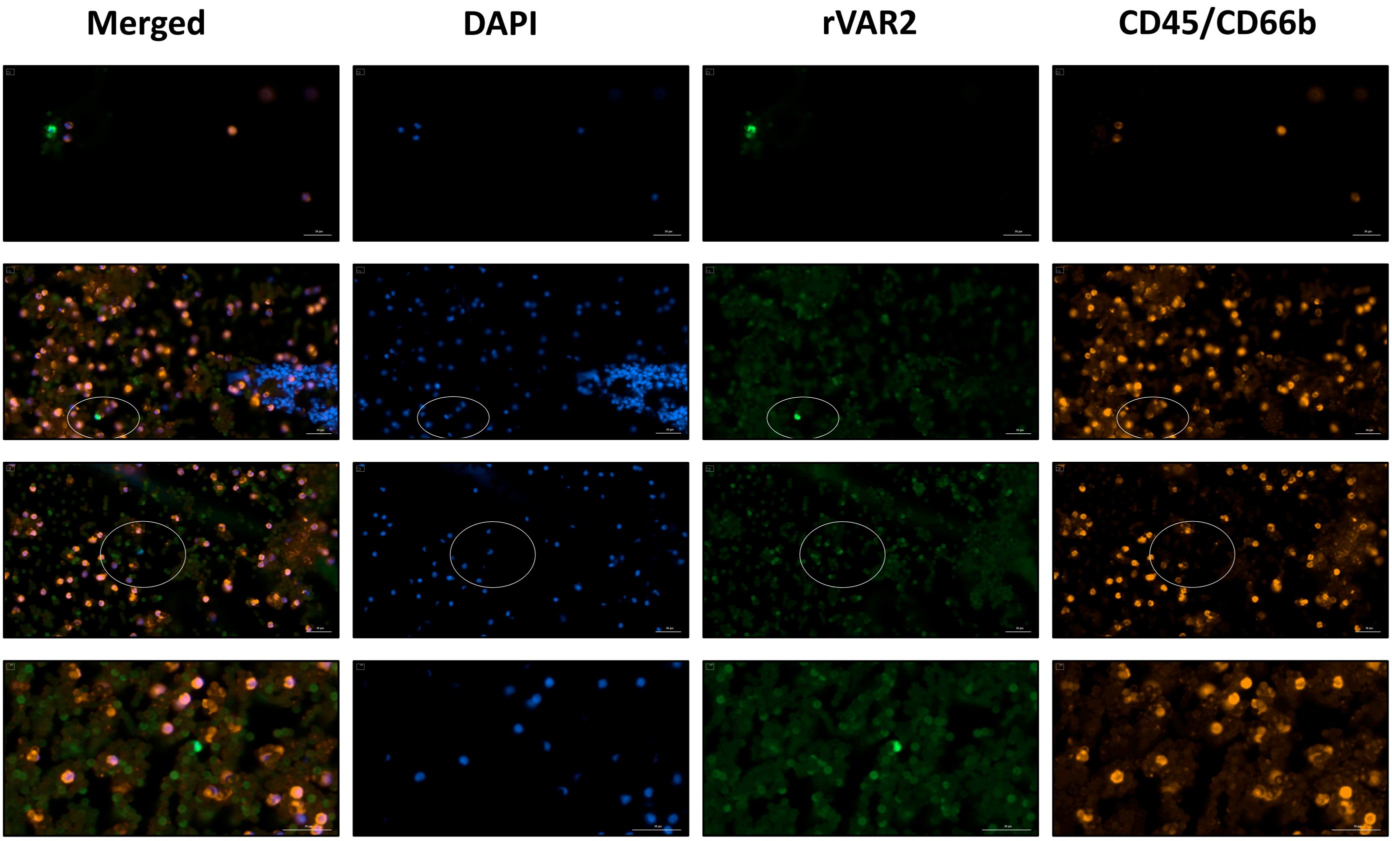

Patient 7, sample B (3mL)

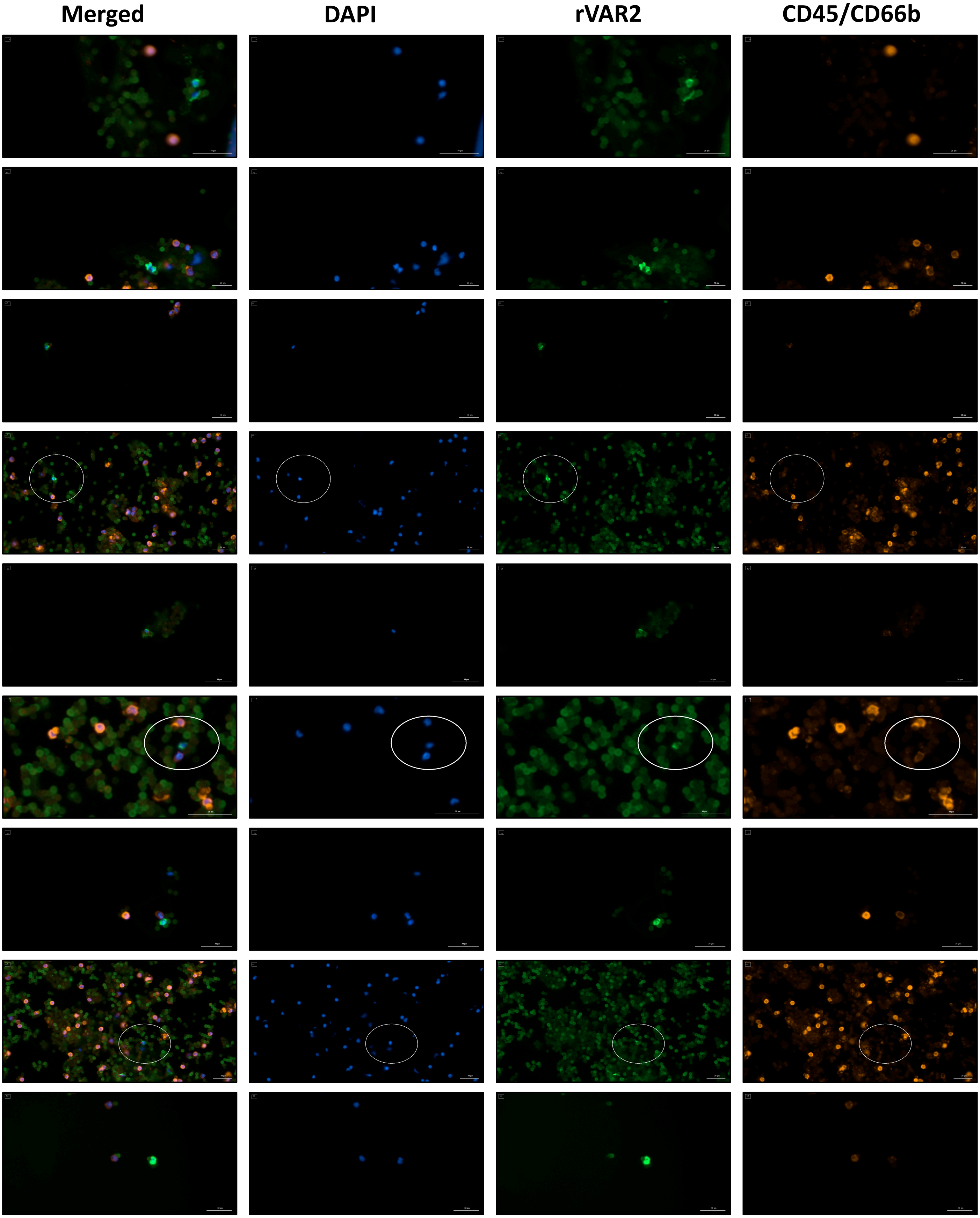

Patient 8, sample A (3mL)

Merged **DAPI** **rVAR2** **CD45/CD66b**

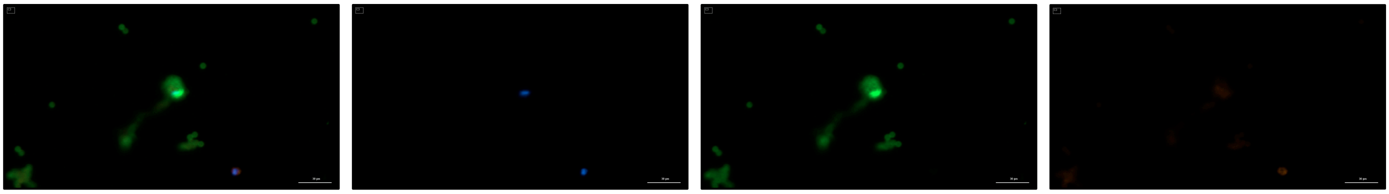

Patient 8, sample B (3mL)

Merged | **DAPI** | **rVAR2** | **CD45/CD66b**

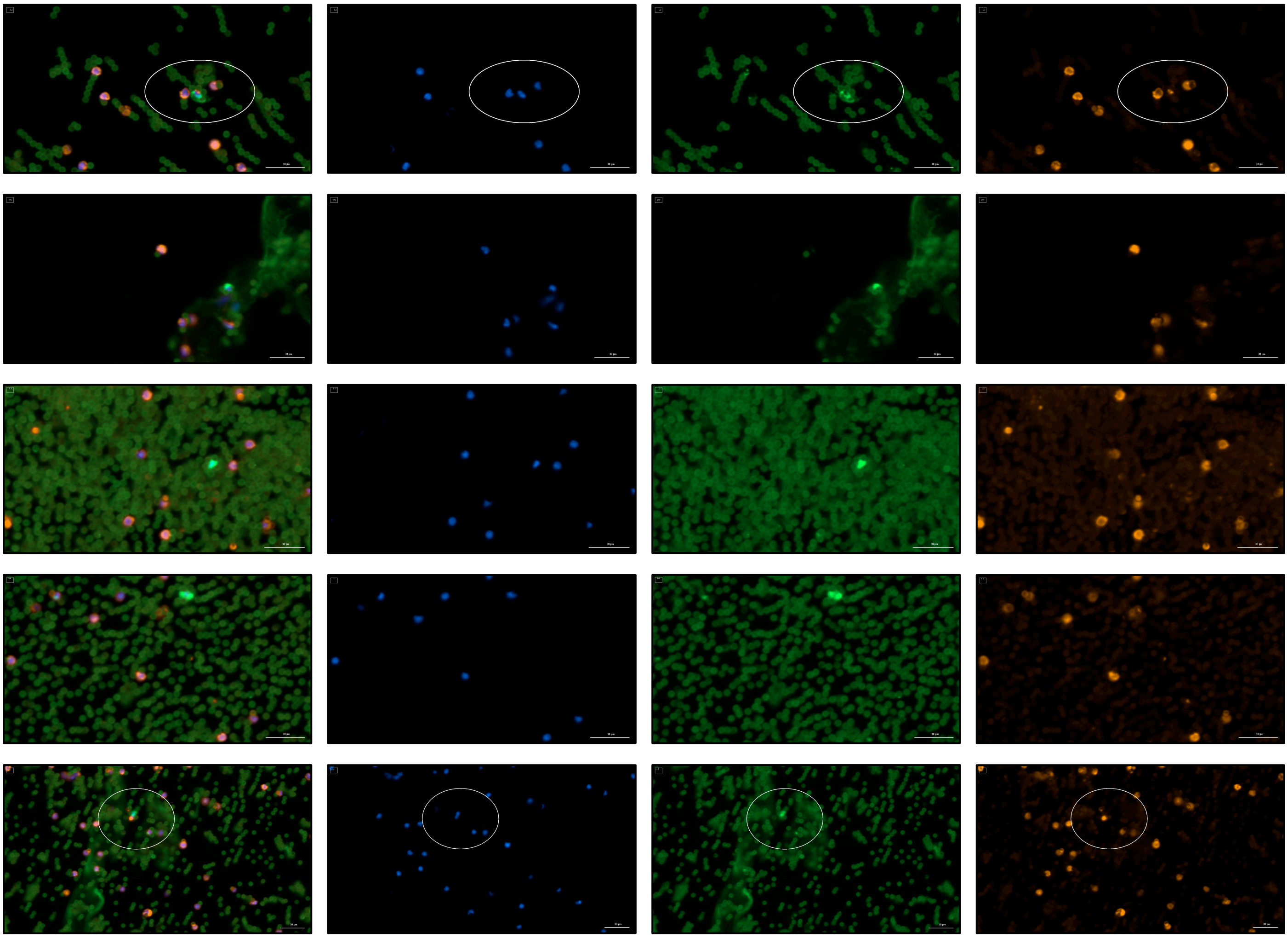

Patient 9, sample A (3mL)

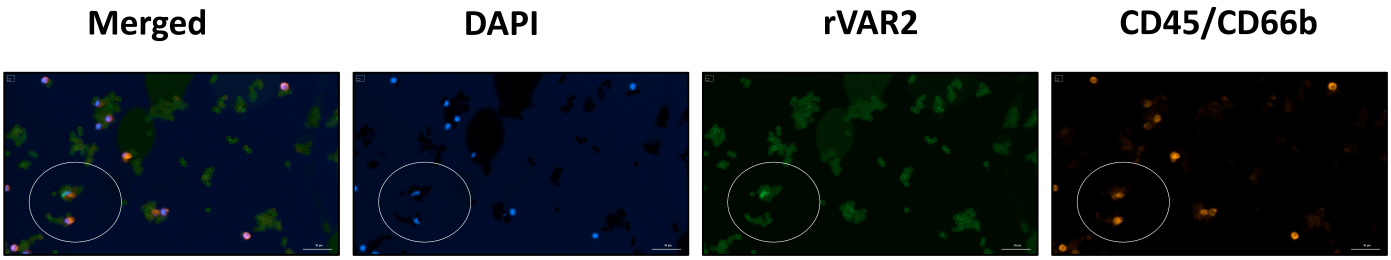

Patient 10, sample A (3mL)

Merged **DAPI** **rVAR2** **CD45/CD66b**

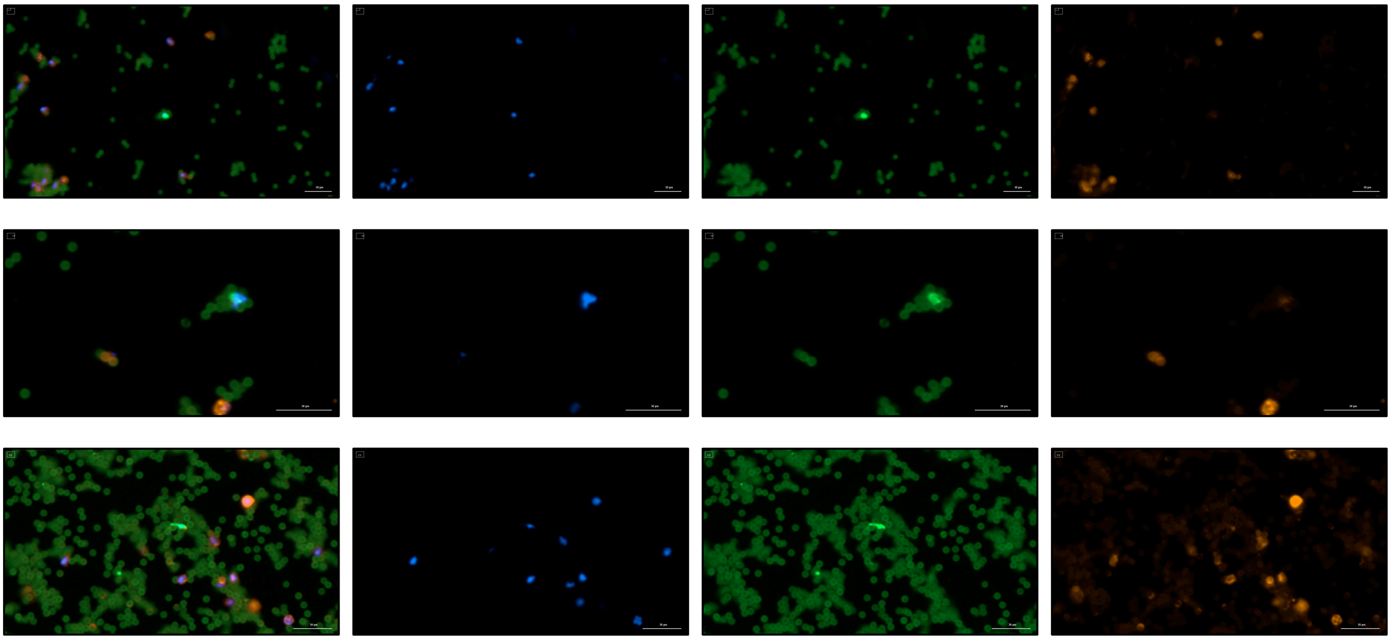

Patient 10, sample B (3mL)

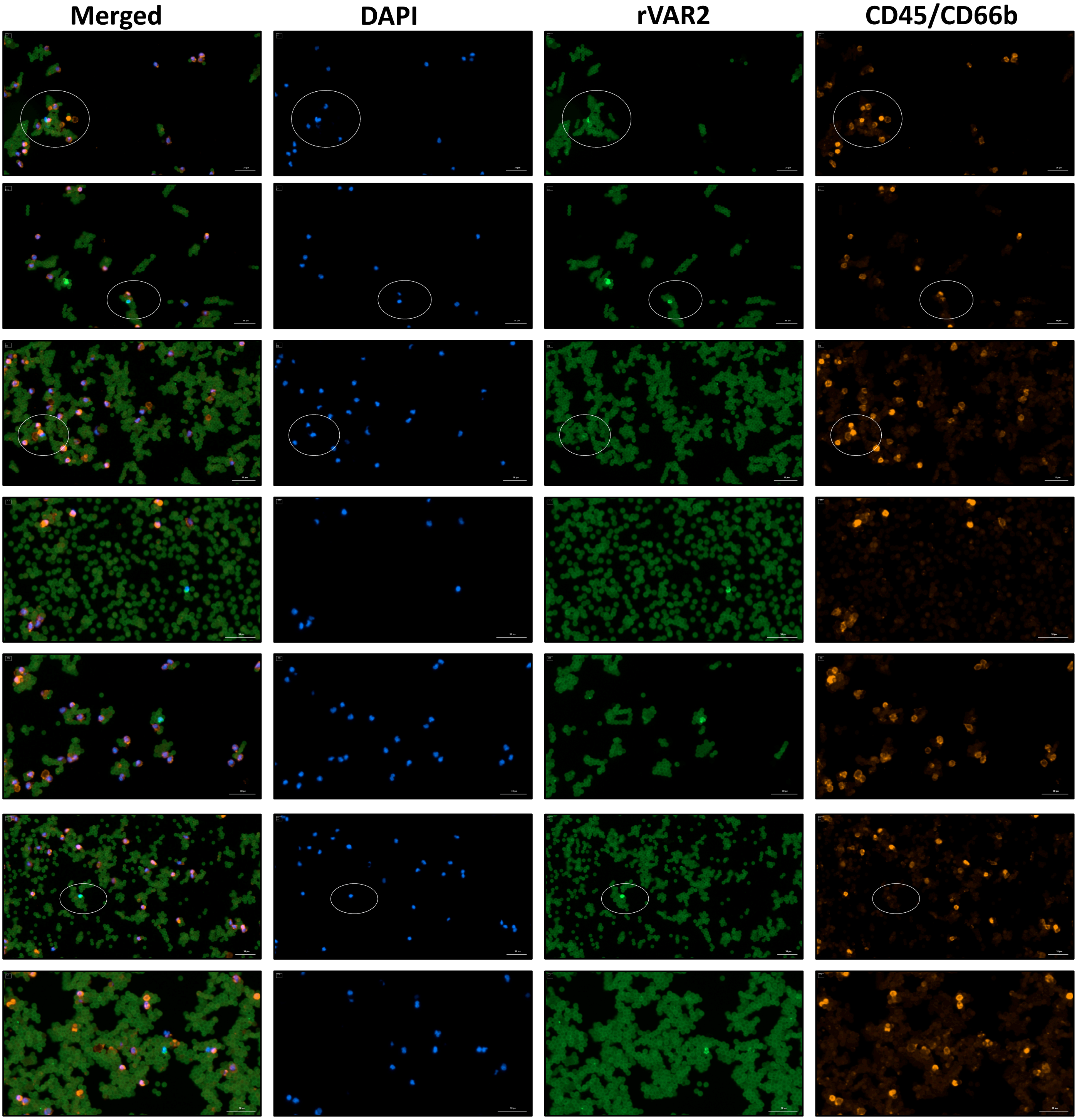

Patient 10, sample B (3mL)

Merged **DAPI** **rVAR2** **CD45/CD66b**

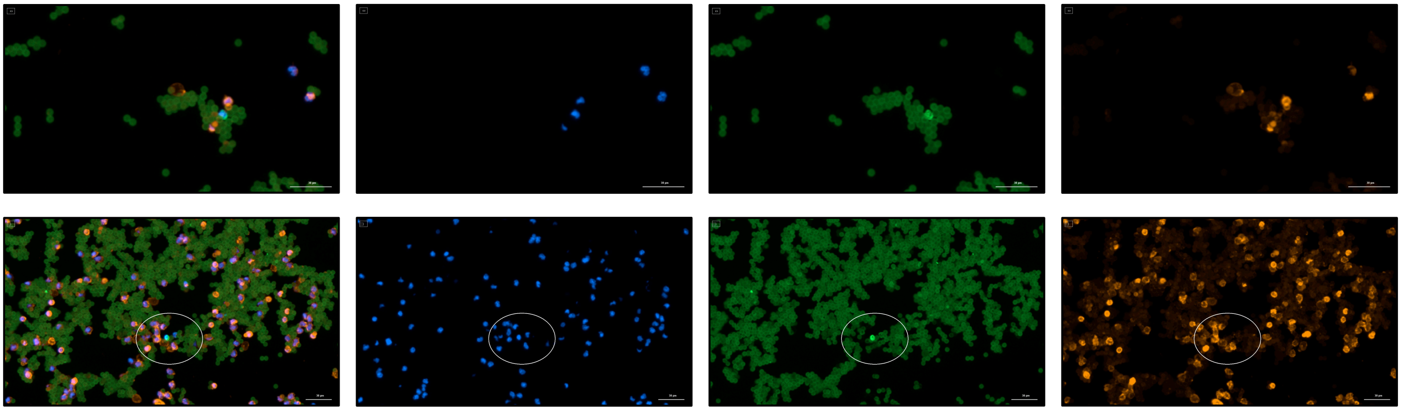

Supplement: Supplementary file 1 [file cells-08-00998-s001.zip › SUPPLEMENTARY FIGURES 1-5_resub PDF.pdf]
