# Supplementary material for: Capture and Detection of Circulating Glioma Cells Using the Recombinant VAR2CSA Malaria Protein
Source: Cells. 2019 Aug 28;8(9):998. doi: 10.3390/cells8090998 (PMC6769911; doi:10.3390/cells8090998)

Supplementary figure S6. *In vitro* EMT western blot

Uncropped images of western blots presented in figure 1B. Res259 cell lysates after 72 hour incubation with TGF-β or buffer control. Membranes were incubated with anti-β-catenin (92 kDa), anti-N-cadherin (140kDa), anti-vimentin (57 kDa) or anti-GAPDH (37 kDa) antibodies and detected by anti-rabbit HRP antibody. Detection of GAPDH served as a loading control.

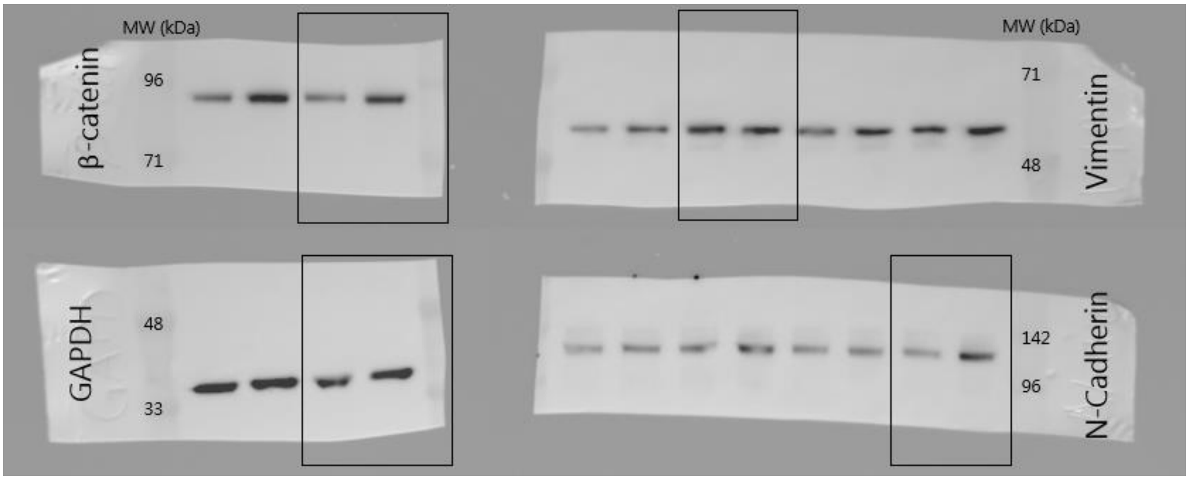

Supplement: Supplementary file 1 [file cells-08-00998-s001.zip › SUPPLEMENTARY FIGURES 6_resub.pdf]
